# De novo antioxidant peptide design via machine learning and DFT studies

**DOI:** 10.1038/s41598-024-57247-z

**Published:** 2024-03-18

**Authors:** Parsa Hesamzadeh, Abdolvahab Seif, Kazem Mahmoudzadeh, Mokhtar Ganjali Koli, Amrollah Mostafazadeh, Kosar Nayeri, Zohreh Mirjafary, Hamid Saeidian

**Affiliations:** 1 Department of Chemistry, Science and Research Branch, Islamic Azad University, Tehran, Iran; 2 Dipartimento di Fisica, Universita’ di Padova, Via Marzolo 8, 35131 Padua, Italy; 3 Department of Chemistry, University of Turin, Via Pietro Giuria 7, 10125 Turin, Italy; 4 Department of Organic Chemistry and Oil, Faculty of Chemistry, Shahid Beheshti University, Tehran, Iran; 5 Department of Chemistry, University of Kurdistan, Sanandaj, Iran; 6 Cellular and Molecular Biology Research Center, Health Research Institute, Babol University of Medical Sciences, Babol, Iran; 7 Student Research Committee, Babol University of Medical Sciences, Babol, Iran; 8 Department of Science, Payame Noor University (PNU), PO Box: 19395-4697, Tehran, Iran

**Keywords:** Antioxidant peptides, De novo design, Deep learning, Machine learning, DFT calculation, Molecular dynamics simulations, Peptides, Medicinal chemistry, Organic chemistry, Chemical synthesis, Theoretical chemistry

## Abstract

Antioxidant peptides (AOPs) are highly valued in food and pharmaceutical industries due to their significant role in human function. This study introduces a novel approach to identifying robust AOPs using a deep generative model based on sequence representation. Through filtration with a deep-learning classification model and subsequent clustering via the Butina cluster algorithm, twelve peptides (**GP1–GP12**) with potential antioxidant capacity were predicted. Density functional theory (DFT) calculations guided the selection of six peptides for synthesis and biological experiments. Molecular orbital representations revealed that the HOMO for these peptides is primarily localized on the indole segment, underscoring its pivotal role in antioxidant activity. All six synthesized peptides exhibited antioxidant activity in the DPPH assay, while the hydroxyl radical test showed suboptimal results. A hemolysis assay confirmed the non-hemolytic nature of the generated peptides. Additionally, an in silico investigation explored the potential inhibitory interaction between the peptides and the Keap1 protein. Analysis revealed that ligands **GP3**, **GP4**, and **GP12** induced significant structural changes in proteins, affecting their stability and flexibility. These findings highlight the capability of machine learning approaches in generating novel antioxidant peptides.

## Introduction

Low concentrations of free radicals play a vital role in various human biological functions, including apoptosis and protein phosphorylation processes^1^. However, an excess of free radicals in the human body is frequently associated with diseases such as Alzheimer, cancer, and other chronic disorders^2,3^. Hence, it is imperative to discover safe, efficient, and readily available antioxidants to prevent the harmful effects of free radicals on cells or reduce their negative impact. Peptides emerge as a favored option for meeting this demand due to their uncomplicated synthesis methods in comparison to other alternatives and also can exhibit additional beneficial properties such as antimicrobial and anticancer activities^4–6^.

**Figure 1:**
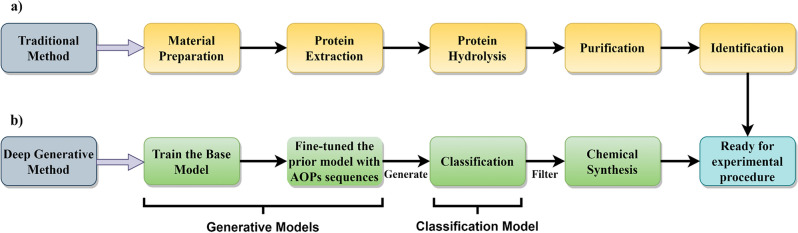
The traditional and the de novo generative method pipelines for antioxidant peptide design.

Nonetheless, material preparation, protein extraction, hydrolysis, purification, and identification of antioxidant peptides (AOPs) can be a time-consuming and challenging endeavor (Fig. 1a)^7–12^. Regarding AOPs, data collected by Olsen et al.^13^ indicates the existence of just 696 such peptides from various natural sources. This disparity underscores the underexplored chemical space of antioxidant peptides, primarily due to the intricate nature of their identification process. To address this challenge, machine learning models offer a promising alternative to traditional methods for rapidly discovering compounds with desired properties, as demonstrated by recent advancements in using ML for anticancer and antimicrobial peptides and food chemistry^14–17^. Consequently, in this research, for the first time, we have developed a generative model based on recurrent neural networks (RNNs) for the de novo design of novel AOPs (Fig. 1b).

Given the relatively small dataset of 696 unique sequences in the peptide antioxidant dataset, training a generative model from scratch was impractical. Therefore, we initially trained our RNN model using a dataset of peptide sequences compiled by Specht et al.^18^. This choice was motivated by the similarity in the distribution of key amino acids between the antioxidant peptides and this dataset.

**Figure 2:**
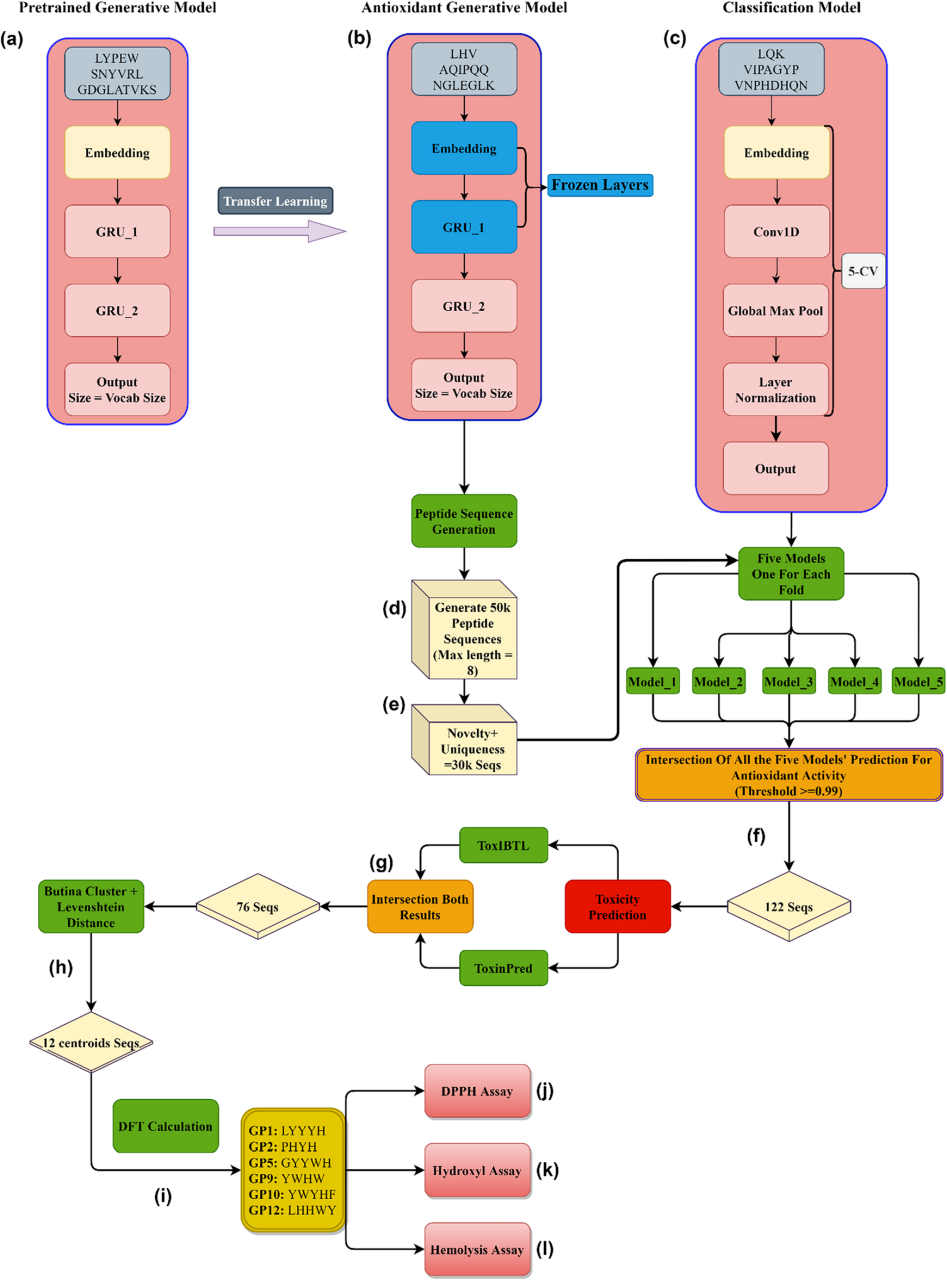
An overview diagram for the de novo antioxidant peptide design. (a) The base (pre-trained) generative model. (b) The fine-tuned model for predicting AOPs. (c) Classification model for predicting the antioxidant activity of the generated sequences. Five models were developed from the fivefold classification evaluation. (d) 50 thousand peptide sequences with a maximum of eight amino acids were generated from the fine-tuned model. (e) Filtering the generated peptides based on their novelty and uniqueness. (f) Filtering the remaining generated peptides by using antioxidant classification models and intersecting the results with thresholds of 0.99 and greater based on the output probability of all of the five classification models. (g) Intersection of two peptide toxicity prediction web servers on the 122 remaining peptide sequences. (h) Clustering the remaining sequences with Levenshtein distance and choosing the centroids’ data points of each cluster. (i) Implementing the DFT calculations on the twelve peptides and selecting six peptides based on their properties. (j) Implementing DPPH scavenging assay. (l) Implementing hydroxyl scavenging assay. (k) Implementing hemolysis assay.

Subsequently, we employed transfer learning to fine-tune our model specifically for generating AOPs. All obtained data from the fine-tuned model underwent classification to determine their scavenging activity. This classification relied on a model trained on the antioxidant dataset, along with assessments of their toxicity from two distinct servers. After this rigorous filtering process, a final list of peptides was compiled. These peptides were further analyzed by clustering their sequences, and the centroid peptides were selected for subsequent investigation. Density functional theory (DFT) calculations were employed to assess molecular properties, including the highest occupied molecular orbital (HOMO), the lowest unoccupied molecular orbital (LUMO), and the HOMO–LUMO energy gap^19–22^. These parameters served as criteria for the antioxidant ranking of the peptides in the present study. Based on these calculated parameters, we selected six peptides with a refined set of candidates for further investigation. Subsequently, we have demonstrated that by synthesizing and testing the chosen peptides, we will be able to identify the non-hemolytic AOPs using 2,2-diphenyl-1-picrylhydrazyl(DPPH), hydroxyl, and hemolysis assays. All the implemented processes are shown in (Fig. 2).

In our pursuit of robust antioxidant peptides, we embarked on a multifaceted strategy, to discover if the generated peptides could simultaneously exhibit antioxidant scavenging activity and inhibit the Keap1 protein by utilizing molecular dynamic (MD) simulations. The Keap1-Nrf2 protein–protein interaction (PPI) is pivotal in regulating Nrf2, a transcription factor that safeguards cells against oxidative stress by controlling the transcription of over 200 antioxidant response element (ARE)-containing genes. The Keap1, in complex with Cullin3-RBX1, negatively regulates Nrf2 through cytosolic binding, promoting its ubiquitination and proteasomal degradation. Oxidative stress, a major contributor to various pathological conditions, underscores the importance of disrupting the Nrf2/Keap1 PPI. This disruption is considered a promising strategy to upregulate Nrf2 levels and enhance cellular protection against oxidative stress^23–25^.

## Materials and methods

### Dataset

To train our pre-trained model, the peptide dataset detailed in Ref.^18^, was utilized which comprises approximately 10,000 distinct sequences. Subsequently, for the development of generative antioxidant models, we turned to the antioxidant peptide dataset as described in Ref.^13^. During the data preprocessing phase, we established a criterion limiting peptide sequences to a maximum of 15 residues. For fine-tuning the generative model, peptides classified as non-antioxidant and chelators were deliberately excluded. Likewise, for the classification model, we specifically excluded chelator peptides to focus exclusively on evaluating the scavenging antioxidant activity of peptides (Fig. 2).

### Generative model

The generative models were developed by using Tensorflow^26^ (Fig. 2). Leveraging the natural-based amino acid dataset at our disposal, characterized by a vocabulary size of 20, our workflow involved tokenizing and encoding input sequences, and following by their input into the embedding layer. Subsequently, the embedding outputs were fed to two gated recurrent units (GRUs) layers^27^. The final layer was a dense layer with an output shape matching the vocabulary size, augmented with a softmax function. During training, we employed Sparse Categorical Cross Entropy as the loss function, closely monitoring validation loss to select the optimal model throughout the training process. To ensure robustness, 90% of the base dataset was allocated for training, while the remaining 10% served as a validation set. The base model underwent 250 epochs of training, with Adam as the optimizer^28^.

In the context of creating an antioxidant generative model, we implement a transfer learning approach. This technique involves transferring knowledge acquired by the model from a larger dataset, containing more general information about peptide sequences and their representation, to train a model for a specific task with a smaller dataset. This approach led to improved performance compared to deploying an untrained model, as deep learning models typically struggle with limited datasets. Initially, we employed the same base model trained on the larger dataset, preserving the embedding layer and the first GRU layer (Fig. 2). However, we made a deliberate decision to unfreeze the second GRU layer and the final dense layer, enabling them to learn the nuances of grammar and connections within antioxidant peptide sequences. Parallel to the base model, we used 90% of the antioxidant peptide sequences for training, with the remaining data serving as the validation set. Again, the training process relied on the Sparse Categorical Cross Entropy loss function, with Adam as the optimizer. Notably, the antioxidant generative model underwent an extensive training regimen of 10,000 epochs, setting it apart from the base model (ESI File, S1).

### Classification model

For the classification task, our workflow involved tokenizing and encoding the input sequences, mirroring the process used for the generative models. The resulting vectorized data was then passed through an embedding layer, followed by a one-dimensional convolutional layer. Subsequently, the output underwent global max-pooling and a layer normalization step to ensure the data’s consistency before reaching the final layer. The concluding layer in our model is a dense layer, characterized by an output shape of one and a sigmoid activation function. This choice aligns with the binary nature of our classification task. To address the challenge posed by the disparity in the number of data points per label within the dataset, we opted to evaluate our model’s performance using the AUC-ROC metric during training across each fold of the fivefold cross-validation process (Fig. 2c). This approach was chosen because, in the presence of the imbalanced dataset, accuracy alone can be misleading and might result in overfitting. Nonetheless, we closely monitored both accuracy and precision throughout the cross-validation process. For training, the Adam optimizer and Binary Cross Entropy as the loss function were utilized (ESI File, S1).

### In-silico toxicity prediction and clustering

The ToxIBTL and ToxinPred, two peptide toxicity prediction servers, were employed to ensure that the final generated structures contained non-toxic peptides ^29–31^. We specifically selected sequences that were predicted as non-toxic by both servers. To group the sequences, we applied the Butina cluster algorithm from RDKit, utilizing the Levenshtein distance as the clustering parameter (Fig. 2)^32^.

### DFT analysis

All DFT analyses including geometry optimization and electronic structure calculations were performed using the Generalized Gradient approximation (GGA). We have used the Perdew–Burke–Ernzerhof (PBE) functional integrated with Grimme’s approach (PBE–D) using the Dmol3 first-principles package^33^. Our wave functions were generated using a numerically tabulated basis set of double-ζ plus polarization (DNP) quality. In addition, we harnessed DFT semi-core pseudopotentials (DSSP) to effectively describe electron–ion interactions through a unified potential. We further utilized the BLYP exchange–correlation functional to support the data. Vibrational frequency evaluations were carried out for the twelve optimized peptide structures, designated as **GP1–GP12**, to ascertain that each structure is in a stable state. No imaginary (negative) frequencies were observed in the frequency calculations, confirming that every structure was optimized to its geometric minimum. Furthermore, all the data reported were considered in water as a solvent using the COSMO (Conductor-like Screening Model) methodology^34^. The energy gap (E_g_) between the highest occupied molecular orbital (HOMO) and the lowest unoccupied molecular orbital (LUMO) for **GP1–GP12** was determined.

### Peptide synthesis

All the required Fmoc-amino acids, reactants, and 2-chlorotrityl chloride (2-Cl-Trt) resin for the synthesis of peptides **GP1**, **GP2**, **GP5**, **GP9**, **GP10,** and **GP12** were purchased from GL Biochem and used without further purification. All solvents (DMF, MeOH, DCM, and CH_3_CN) were purchased from Merck ((Darmstadt, Germany) and Fluka (Neu-Ulm, Germany) companies. The used amino acids were as follows: Fmoc-His (Trt)-OH, Fmoc-Tyr (tBu)-OH, Fmoc-Leu-OH, Fmoc-Trp(Boc)-OH, Fmoc-Leu-OH, Fmoc-Pro-OH. The 2-chlorotrityl chloride (2-Cl-Trt) resin (2 g, with loading 0.4 mmol/g) was washed with DCM (2 × 5 min, 15 mL). The resin was loaded with an amino acid using 3 equiv. Fmoc-protected amino acid and 6 equiv. of diisopropylethylamine (DIEA) in DCM (20 mL) at room temperature for 2 h. After reaction completion, the solid was filtered, and added to a mixture of MeOH, DCM, and DIEA (83/11/6, v/v/v, 3 × 10 min, 20 mL), and the loaded resin was washed with DMF (2 × 5 min, 15 mL), DCM (1 × 5 min, 15 mL) and DMF (2 × 5 min, 15 mL). Before the next coupling reaction for the second amino acid, Fmoc deprotection was conducted with 30% of piperidine in DMF (2 × 5 min, 10 mL) and filtration and washing the solid with DMF (2 × 5 min, 15 mL), the next amino acid was coupled using the standard solid phase peptide synthesis protocols^35^ and this procedure repeated for other amino acids in our peptide sequence. For cleavage of the desired peptide from the 2-Cl-Trt resin and removal of protective groups from the side chain groups, a mixture of trifluoroacetic acid, phenol, thioanisole, triisopropylsilan, and water (83/5/5/2/5, v/v/v/v/v, 45 mL) was added to the resin and stirred for 2 h and then filtered. The crude peptide was precipitated in ether, filtrated, and washed with ether (3 × 30 mL), followed by vacuum drying overnight. Molecular mass and purity of the six synthesized peptides were determined by LC–MS method using an Agilent 1200 LC system (Agilent, Waldbronn, Germany) coupled to an Agilent 6410 triple quadrupole tandem mass spectrometer (Agilent Technologies, CA, USA).

### DPPH scavenging activity assay

A straightforward and expeditious approach for quantifying the antioxidant activity of antioxidants involves employing the 2,2-diphenyl-1-picrylhydrazyl (DPPH) assay, a spectrophotometric technique^36–38^. Preparation of the test sample, reagent, and control sample in the DPPH assay is as follows: peptide samples with a concentration of 10 mg/mL were prepared in ethanol and then were diluted in 2, 4, 6, and 8 mg/L concentrations. The DPPH solution is prepared with a concentration of 0.02% (w/v) in methanol. A solution of 0.5 mM ascorbic acid has been prepared as a positive control, and methanol is considered as the negative control sample. The peptide sample (250 μL) with DPPH solution (250 μL) in ethanol (1.0 mL) was mixed and stirred in the dark for 30 min at room temperature. The solutions were analyzed using a UV–Vis instrument (Varian, Cary 100) at 517 nm. The calculation of antioxidant activity percentage is reported as the following equation:

1 $${\text{DPPH scavenging activity }}\% \, = {\text{ A}}_{0} - {\mathrm{A}}_{{1}} /{\mathrm{A}}_{0} \times { 1}00.$$

In Eq. (1), A_0_ is the absorbance of the negative control (methanol) and A_1_ is the optical absorbance of the peptide samples.

### Hydroxyl scavenging activity assay

Hydroxyl radical is one of the strong reactive oxygen species in biological systems that react with the unsaturated fatty acid of phospholipids of the cell membrane and cause cell damage^36–38^. To assess the efficacy of peptides in countering hydroxyl radicals, we subjected them to a hydroxyl radical-scavenging activity assay, indicating their scavenging potential across a range of concentrations. The method for preparing the test samples, reagents, and control samples for the hydroxyl scavenging activity assay is as follows: initially, peptide samples at 10 mg/mL concentration were dissolved in ethanol, and subsequently, diluted concentrations of 2, 4, 6, and 8 mg/L were obtained from this solution. The assay reagent includes 6 mM hydrogen peroxide and 6 mM ferrous sulfate in ethanol. A solution of ascorbic acid with 0.5 mM concentration has been prepared as a positive control and a solution without the peptide sample is considered as negative control. A colored solution was obtained by adding 200 μL of the assay reagent and 200 μL of the peptide sample. The mixture was shaken for 10 min at room temperature. Then 200 μL of 6 mM salicylic acid was added to the mixture. After 30 min, the UV–Vis absorbance was determined at 510 nm. The determination of hydroxyl radical scavenging activity for the peptide samples was executed as follows:

2 $${\text{Hydroxyl radical scavenging activity }}\% \, = {\text{ A}}_{0} - {\mathrm{A}}_{{1}} /{\mathrm{A}}_{0} \times { 1}00,$$

where A_0_ is the absorbance of the negative control (solution without the peptide sample) and A_1_ is the absorbance of the peptide samples.

### Hemolysis assay

The hemolysis test was performed as standard protocol. A 1.5 mL heparin blood sample was obtained from a healthy volunteer in our laboratory. The red blood cells (RBCs) were collected by centrifugation of blood samples at 3000 rpm for 15 min and then were washed three times with PBS. The RBC pellet was resuspended in a 10 mL phosphate-buffer solution (PBS). A 100 µL/well of different concentrations of peptides were added into a 96-well plate (2000 µg/mL, 1000 µg/mL, 500 µg/mL, 250 µg/mL, 125 µg/mL, 62.5 µg/mL, 31.25 µg/mL, 15.62 µg/mL, 7.8 µg/mL). For positive hemolysis control 100 µL/well of 0.1% sodium dodecyl sulfate and distilled water, and for negative hemolysis control 100 µL/well of PBS were added into the appropriated wells. After that, 100 µL RBC suspension was added into all the wells. The plate was incubated at room temperature for 4 h. Then the 100 µL supernatant of each well was carefully transferred to a new 96-well plate. The solution absorbance was investigated by a UV–Vis instrument at 450 nm.

### Molecular dynamics analysis for Keap1 protein

Molecular dynamic (MD) simulations were conducted on 13 different systems. The reference system consisted of a receptor, water, and the appropriate amounts of sodium and chloride ions to achieve a salt concentration of 0.15 M; additionally, the other 12 systems included a receptor, water, salt, and one of each of the peptides **GP1–GP12**. The MD interaction was taken in place in the active site of the KEAP1 protein. To construct all the simulation systems, CHARMM-GUI was utilized^39–41^. The simulations were performed in the NPT ensemble using the GROMACS 5.1.5 simulation package^42–44^. The CHARMM36m force field^45,46^ was applied to both the ligands and receptor. Temperature (310 K) and pressure (1 bar) were maintained during the simulations. Temperature control was achieved using the Nose–Hoover thermostat^47^ with a coupling time of 0.5 ps, while pressure control was accomplished by coupling the simulation cell to a Parrinello–Rahman barostat with a coupling time constant of 2 ps^48^. Periodic boundary conditions were employed, and the transferable intermolecular potential 3-point (TIP3P) water model was used^49^. Atom bond lengths were constrained using the LINCS algorithm^50^. The equations of motion were integrated using the leap-frog algorithm with a time step of 2 fs^51^. Coulomb and van der Waals interactions were cut-off at 1.2 nm, and long-range electrostatic interactions were handled using the particle mesh Ewald method^52^. Unfavorable atomic contacts were eliminated through the steepest descent energy minimization^53^. Initially, the positions of the ligands were restrained, and equilibration was performed in the NVT ensemble for 1 ns, followed by equilibration in the NPT ensemble for 9 ns. After the equilibration steps, all simulations were run for 250 ns starting from their initial conditions and atom coordinates.

### Ethical approval

The authors have fully observed the ethical points in conducting the research and writing the results. All methods were carried out in accordance with relevant guidelines and regulations. All experimental protocols were approved by the Payame Noor University Research Committee. Informed consent was obtained from all subjects and/or their legal guardian(s).

## Results and discussion

### Deep generative model

**Figure 3:**
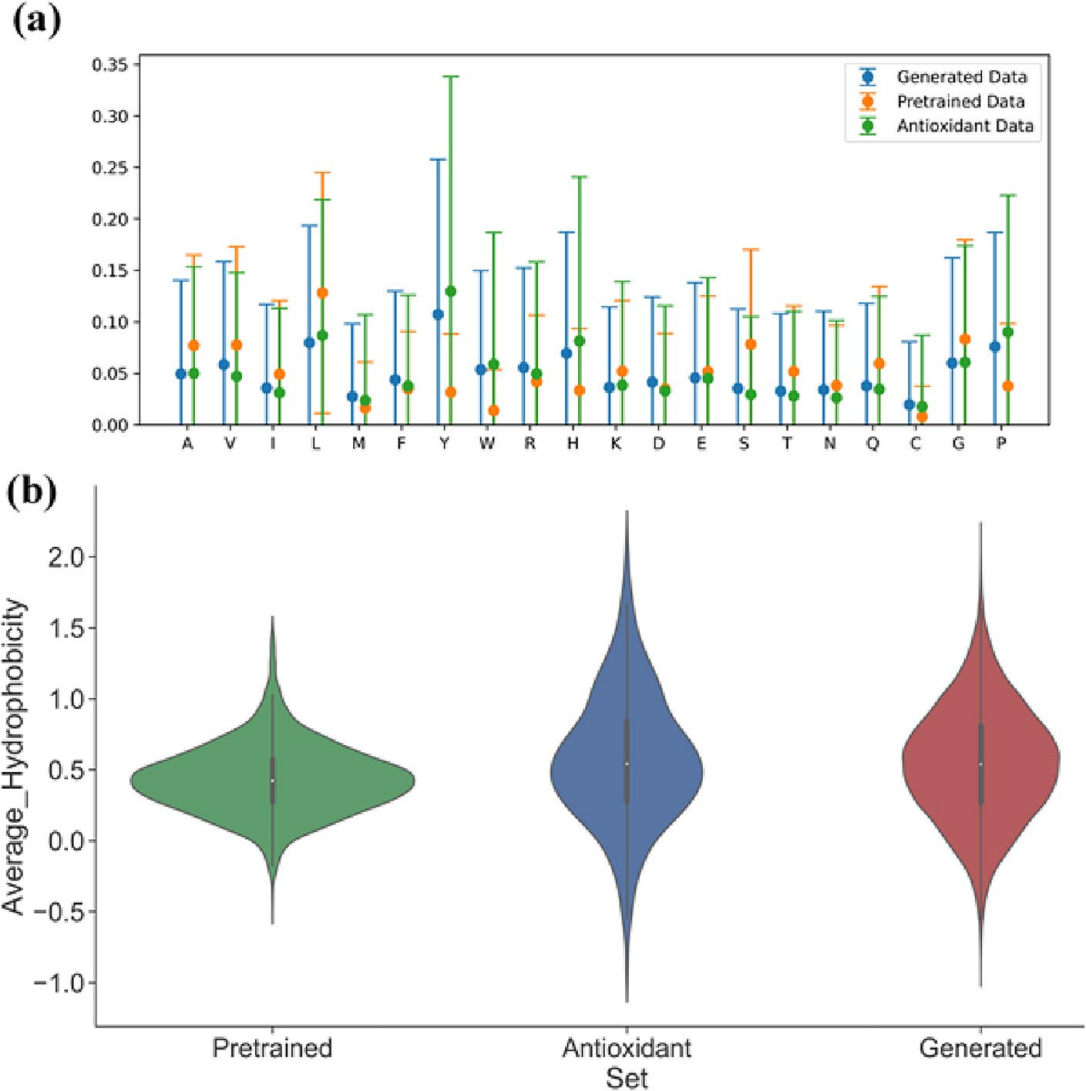
Chemical space analysis and comparison of the pre-trained, antioxidant, and generated datasets. **(a)** Mean amino acid fractions and their standard distribution of the three datasets that were used and generated. **(b)** Average hydrophobicity of the pre-trained, AOPs and the AOPs generated dataset.

By utilizing the antioxidant generative model, 50 k peptide sequences were generated (Fig. 2f). In line with our predetermined criteria, the length of these peptides was restricted to a maximum of eight amino acids. To make sure that we only analyze and work with novel and unique peptide sequences, we remove these redundant sequences, thus ensuring that each peptide entry in our dataset is unique. Furthermore, for the elimination of any duplications, an analysis within the pre-existing peptide database was conducted^54^. Through these procedures, we successfully created a collection of nearly 30k unique and novel peptide sequences (as illustrated in Fig. 2e). It is noteworthy that the uniqueness of this curated dataset, in which the peptide length was capped at eight amino acids, was quantified at 61.8%, reflecting the challenges posed by the imposed constraint. However, the novelty of the generated sequences was notably high, with a 97.3% novelty score. To validate the thorough understanding of AOP representations by our fine-tuned model, a comprehensive analysis of the generated peptide sequences was conducted. This analysis encompassed an examination of their average hydrophobicity, mean amino acid fraction, and standard deviation across multiple datasets, including the AOP dataset used for fine-tuning, as well as the pre-trained dataset. Our findings affirm that the antioxidant model adeptly acquired the amino acid representations essential for the task at hand, evident in its ability to generate peptides closely aligned with the characteristics of the AOP dataset (Fig. 3).

### Antioxidant classification model

To enhance the rigor of our approach, a fivefold classification process was applied which resulted in preserving all generated models for subsequent analysis. The model achieved an average ROC-AUC score of 82.64% and accuracy scores reaching 76.47%, 76.53% for precision, and 52.88 for The Matthews correlation coefficient (MCC). These metrics are summarized in Table 1. To assess the activity of generated sequences, we employed all five models from each fold to predict whether a peptide possesses antioxidant activity. For a peptide to qualify, it must receive unanimous approval from all five models, with a threshold set at 0.99. In other words, the peptide is accepted only if all five models concur with 99% confidence or higher regarding its antioxidant attributes. Following this methodology, 122 peptide sequences with the desired criteria were identified (Fig. 2f).

**Table 1:** The antioxidant peptide classification model’s performance and the average results.

Model	Accuracy%	Precision%	AUC-ROC%	MCC%
Model fold 1	77.81	79.71	83.04	55.67
Model fold 2	77.09	74.52	83.8	54.48
Model fold 3	77.09	76.37	83.02	54.0
Model fold 4	74.45	77.67	81.03	48.74
Model fold 5	75.91	74.4	82.31	51.52
Average	76.47	76.53	82.64	52.88

### Toxicity prediction and clustering

The remaining peptides were uploaded to the ToxinPred and ToxIBTL web servers. We intersected those results and only selected the peptides considered nontoxic by both web servers. In this step, 76 peptides remained for the next steps (Fig. 2g). In the subsequent stage, we applied clustering to the filtered sequences using the RDKit Butina module, employing a threshold of 2 and utilizing the Levenshtein distance as the distance function. From each cluster, we selected the central sequence. This workflow introduced a total of 12 peptides (Fig. 2h).

### DFT analysis

**Figure 4:**
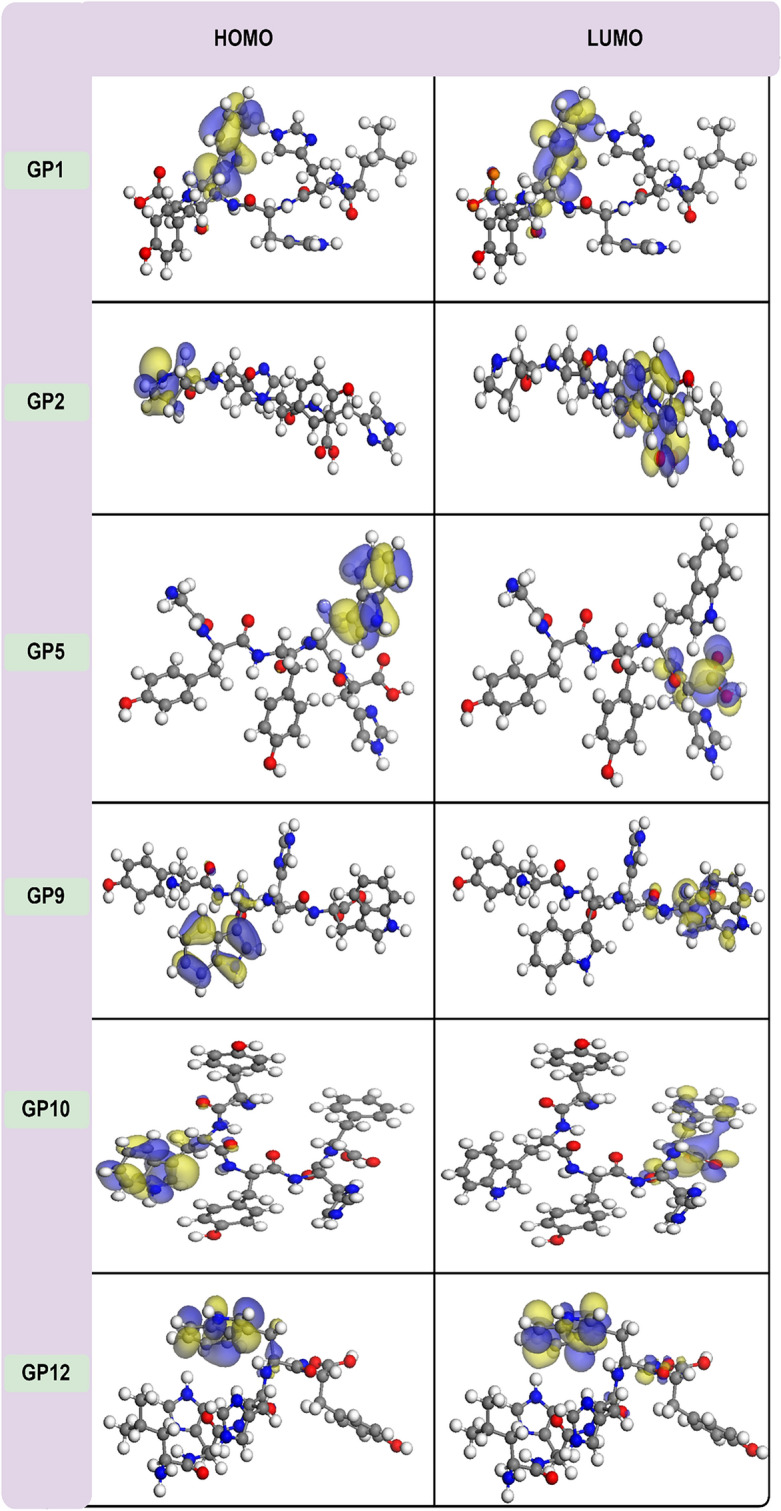
The HOMO and LUMO maps for the six synthesized peptides. The red ball represents the oxygen atom; the light gray ball represents the hydrogen atom; the dark gray ball represents the carbon atom; the blue ball represents the nitrogen atom.

The ML model proposes twelve unique peptides (**GP1–GP12**), each denoted by a specific sequence (**GP1** (LYYYH), **GP2** (PHYH), **GP3** (AHHHW), **GP4** (HWHYL), **GP5** (GYYWH), **GP6** (LHYYMW), **GP7** (PHYY), **GP8** (YQYYW), **GP9** (YWHW), **GP10** (YWYHF), **GP11** (YYHPF), and **GP12** (LHHWY)). These peptides, yet to be synthesized, exhibit potential antioxidant activity. Experimental validation through synthesis, purification, and in vitro/in vivo analyses is necessary. However, preliminary insight into their antioxidant potential was gained through DFT calculations, leveraging frontier molecular orbital theory. The HOMO and LUMO energies (E_HOMO_ and E_LUMO_) were evaluated, with E_HOMO_ indicative of electron-donating capacity crucial for neutralizing free radicals. Visualization of HOMO, especially in **GP1–GP12**, provides insight into potential attack sites for free radicals (Fig. 4)^55,56^.

Besides, the energy gap (E_g_) between HOMO and LUMO is a pivotal indicator of a molecule’s biological activity. A larger E_g_ signifies increased chemical stability, while a smaller E_g_ suggests enhanced compound polarization and notable electron transfers between donors and acceptors^56^. Moreover, E_HOMO_ and E_LUMO_ energies directly correlate with ionization potential (IP =  − E_HOMO_) and electron affinity (EA =  − E_LUMO_)^57^. A lower IP implies a reduced tendency to lose electrons, signifying a stronger affinity for transitioning to the radical cation form. Practically, this involves removing an electron from the HOMO of the neutral antioxidant, transforming it into the radical cation form. Electron affinity values, as descriptors, express the energy involved in electron abstraction. A higher EA for an antioxidant indicates easier electron abstraction compared to other molecules. Specifically, electrons are assimilated from a free radical into the LUMO of the neutral antioxidant. Table 2, summarizing data for the twelve peptides (**GP1–GP12**) determined by the ML method, reveals noteworthy insights. Particularly, **GP12**, boasting an E_HOMO_ of − 4.88 eV (IP = 4.88 eV), emerges as a potent electron donor, indicative of heightened antioxidant activity. Peptides with lower E_g_ values, exemplified by **GP12**, are anticipated to exhibit diverse interactions with free radicals, potentially intensifying antioxidant efficacy through electron transfer. Conversely, peptides like **GP1** and **GP3**, with higher E_g_ values of 3.67 and 3.68, may be less reactive. Validation of our results using the BLYP exchange–correlation functional underscores their reliability. The E_HOMO_ order of **GP1–GP12**, displaying increasing values from **GP6** to **GP12** (**GP6 = GP11** < **GP7** < **GP1** < **GP2** < **GP4** < **GP3 = GP5** < **GP8 < GP10** < **GP9** < **GP12)**, suggests **GP9**, **GP10**, and **GP12** as potential strong antioxidants due to their higher E_HOMO_ (and lower E_g_). Accordingly, these three peptides, along with **GP1**, **GP2**, and **GP5**, were chosen for synthesis and subsequent antioxidant activity testing using the DPPH and hydroxyl tests (“Antioxidant activity” and “Hemolysis results” sections). HOMO and LUMO mappings for the examined peptides (Fig. 4) highlight the significant influence of the indole ring, particularly in regulating antioxidant actions, through modulation of the HOMO. Notably, instances of coexistence between the LUMO and HOMO underscore the potential for diverse antioxidant mechanisms. This is particularly evident in the case of **GP12**, characterized by the highest HOMO, suggesting a potential for multiple mechanisms, including electron transfer, to contribute to its antioxidant activity.

**Table 2:** The DFT calculated E_HOMO_, E_LUMO_, E_g_, IP, and EA values of the peptides **GP1–GP12**.

Peptides	E_HOMO_	E_LUMO_	E_g_	IP	EA
GP1	− 5.23 (− 5.07)	− 1.56 (− 1.49)	3.67 (3.85)	5.23 (5.07)	1.56 (1.49)
GP2	− 5.21 (− 5.09)	− 1.36 (− 1.32)	3.85 (3.77)	5.21 (5.09)	1.36 (1.32)
GP3	− 5.07 (− 4.93)	− 1.38 (− 1.33)	3.68 (3.60)	5.07 (4.93)	1.38 (1.33)
GP4	− 5.12 (− 4.84)	− 1.48 (− 1.25)	3.64 (3.61)	5.12 (4.84)	1.48 (1.25)
GP5	− 5.07 (− 4.85)	− 1.42 (− 1.27)	3.65 (3.58)	5.07 (4.85)	1.42 (1.27)
GP6	− 5.35 (− 4.90)	− 1.43 (− 1.32)	3.92 (3.60)	5.35 (4.90)	1.43 (1.32)
GP7	− 5.30 (− 5.17)	− 1.47 (− 1.48)	3.63 (3.69)	5.05 (5.17)	1.42 (1.48)
GP8	− 5.06 (− 4.88)	− 1.42 (− 1.31)	3.63 (3.57)	5.30 (4.88)	1.47 (1.31)
GP9	− 4.98 (− 4.80)	− 1.47 (− 1.43)	3.51 (3.37)	4.98 (4.80)	1.47 (1.43)
GP10	− 5.017 (− 4.80)	− 1.45 (− 1.28)	3.56 (3.51)	5.02 (4.80)	1.45 (1.28)
GP11	− 5.35 (− 5.17)	− 1.43 (− 1.42)	3.91 (3.75)	5.35 (5.17)	1.43 (1.42)
GP12	− 4.88 (− 4.74)	− 1.50 (− 1.58)	3.38 (3.16)	4.88 (4.74)	1.50 (1.58)

All the energy units are in eV. In parentheses value calculated by BLYP methods.

The indole ring’s electron-rich nature, highlighted by the HOMO localization, suggests a potential for effective electron donation, aligning with electron transfer mechanisms in antioxidant activity. This, coupled with the conjugated structure, enhances its electron-donating properties, contributing to scavenging reactive oxygen species. Findings align with Trp’s role in antioxidant activity, due to indole rings, emphasizing its hydrogen donation capacity ^57^. However, our HOMO-centric results prompt reconsideration, suggesting a shift to electron transfer mechanisms. The specific role of the indole ring in electron transfer needs careful investigation for refined understanding. This insight adds nuance to the mechanisms governing indole-containing peptides’ antioxidant activities.

### Antioxidant activity

**Figure 5:**
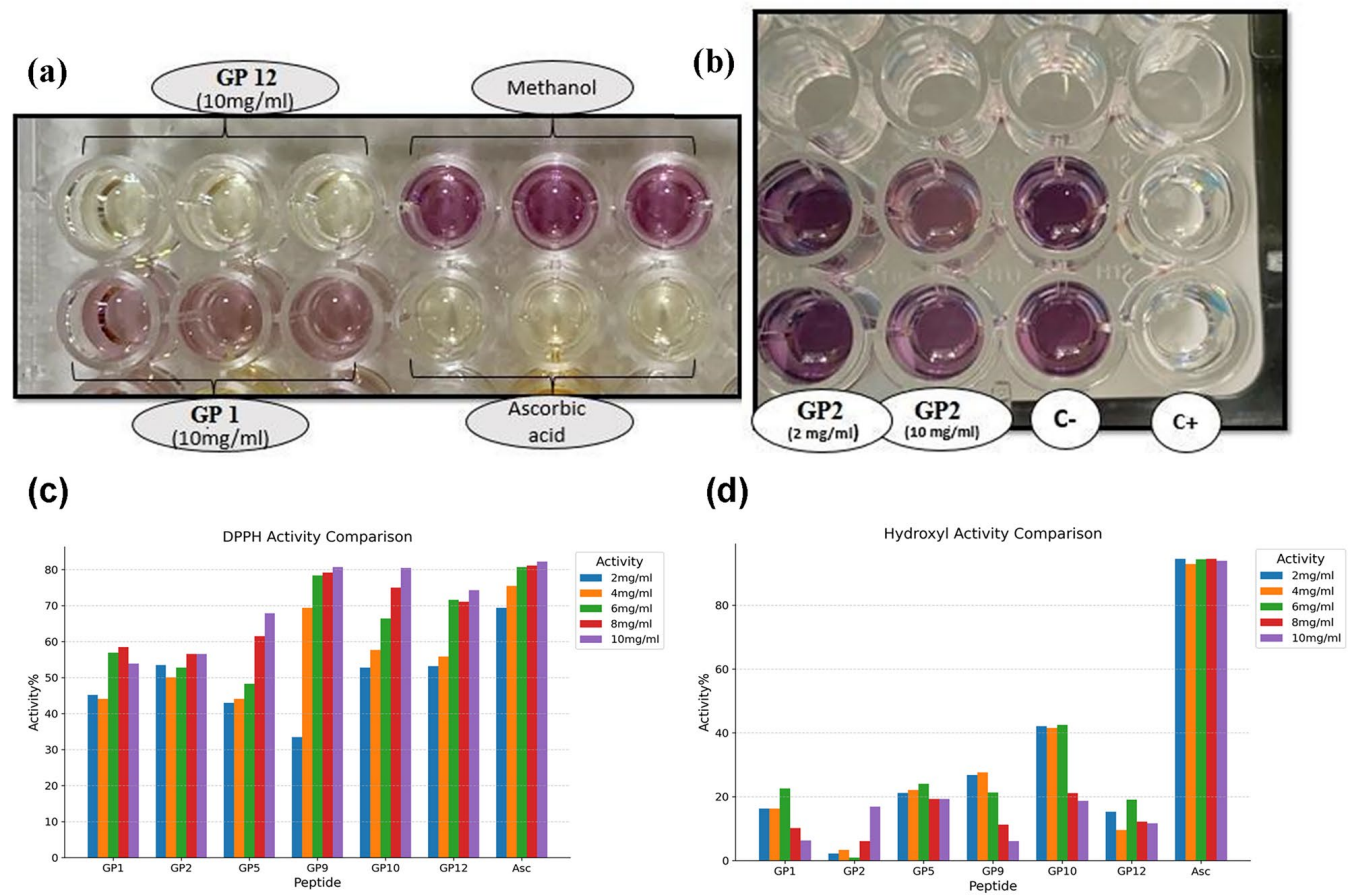
(**a**) Comparison of the color intensity of different samples based on the antioxidant activity in DPPH assay. (**b**) Comparison of the color intensity of different samples based on the antioxidant activity in the hydroxyl radical scavenging activity assay. It should be mentioned that ascorbic acid was used as the positive control (C+) and methanol was used as the negative control (C−). (**c**) The DPPH radical scavenging activity of the six synthesized peptides compared to ascorbic acid (Asc) as a positive control. (**d**) The hydroxyl radical scavenging activity of the synthesized peptides compared to the Asc as a positive control. It should be noted that the concentrations provided refer to those of the stock solution utilized, rather than the final concentrations observed in the assays.

The DPPH assay is based on the reduction of the stable free radical DPPH by accepting electrons from antioxidant which leads to a colored solution^37^. The methanolic solution of DPPH radical has a violet color that shows the maximum light absorption at 519–595 nm. After reduction, the DPPH radical is converted to DPPH2. In this case, the violet color of the solution changes to yellow (Fig. 5a), and absorption intensity at 517 nm decreases. As shown in Fig. 5c, all six synthesized peptides exhibited concentration-dependent free radical scavenging activities. The DPPH radical scavenging activity of the synthesized peptides were 53.9–80.7% at 10 mg/mL. Peptides **GP9**, **GP10,** and **GP12** with the highest E_HOMO_ value show the highest free radical scavenging activity, respectively. These peptides have almost the same antioxidant activity as ascorbic acid. Despite ascorbic acid instability, the peptide-based antioxidants are stable.

In the hydroxyl radical scavenging activity assay, the degree of hydroxyl radical scavenging is measured in different concentrations of the sample (Fig. 5b)^37^. The hydroxyl radical scavenging activities were in the range of 6.1–19.35% at 10 mg/mL (Fig. 5d). In comparison to ascorbic acid (93.9%), the hydroxyl radical scavenging activities of all the synthesized peptides were significantly lower.

### Hemolysis results

**Figure 6:**
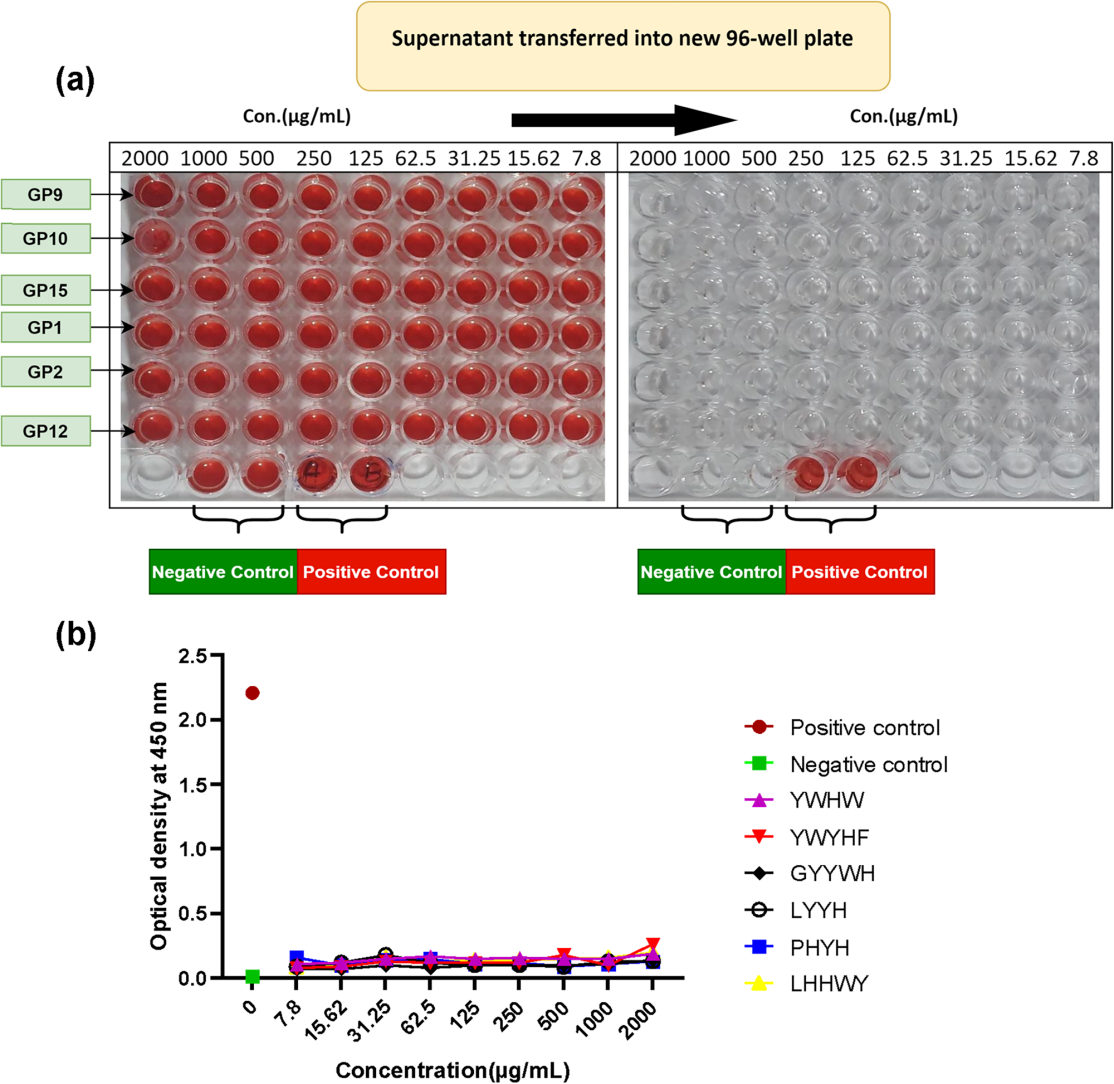
Hemolysis activity of different concentrations of peptides in vitro. The red blood cells were incubated with different concentrations of **GP1**, **GP2**, **GP5**, **GP9**, **GP10**, **GP12**, and 0.1% SDS+ distilled water and PBS were used as positive and negative-control respectively. After 4 h, the supernatants were transferred into a new 96-well plate and the absorbance was read at 450 nm. (**a**) The appearance of supernatant in the wells has shown that, like negative control, there weren’t any hemoglobin particles in wells that were incubated with different concentrations of peptides, but in positive control, the red color of supernatant indicated the release of hemoglobin particles into the supernatant fluid. (**b**) The OD value for negative and positive-control was 0.015 and 2.21 respectively, while the OD of different concentrations of peptides was in a low range, from 0.07 to 0.25.

To evaluate the lytic effect of different concentrations of the synthesized peptides on RBCs, hemolysis assay was conducted. The levels of released hemoglobin into the supernatant fluid were compared with positive and negative controls. As illustrated in Fig. 6a (ESI File, S2) the supernatant of different concentrations of all peptides was clear as well as negative control, whereas in positive control the membrane of RBCs was completely damaged somehow the written letter with a pen were readable obviously (the yellow arrows in Fig. 6a). These letters were not visible in negative control wells. This observation is confirmed by comparison of the optical density (OD) value of the peptide samples with positive and negative control. The OD values for negative and positive control were 0.015 and 2.21 respectively, while the maximum OD value at 0.26 was obtained for RBC which was treated with the highest concentrations of the peptide (2000 µg/mL) (Fig. 6b). The red blood cells were incubated with different concentrations of **GP9**, **GP10**, **GP5**, **GP1**, **GP2**, **GP12** and 0.1% SDS+ distilled water and PBS were as positive and negative control, respectively. After 4 h, the supernatants were transferred into a new 96-well plate and the absorbance was read at 450 nm. Like negative control, the appearance of supernatant with different concentrations of peptides in the wells showed that there weren’t any hemoglobin particles in the wells; but in positive control, the red color of the supernatant indicated the release of hemoglobin particles into the supernatant fluid. The OD value for negative and positive control was 0.015 and 2.21 respectively, while the OD of different concentrations of the synthesized peptides were in a low range, from 0.07 to 0.25.

### Molecular dynamic analysis

#### Root mean square fluctuation (RMSF)

**Figure 7:**
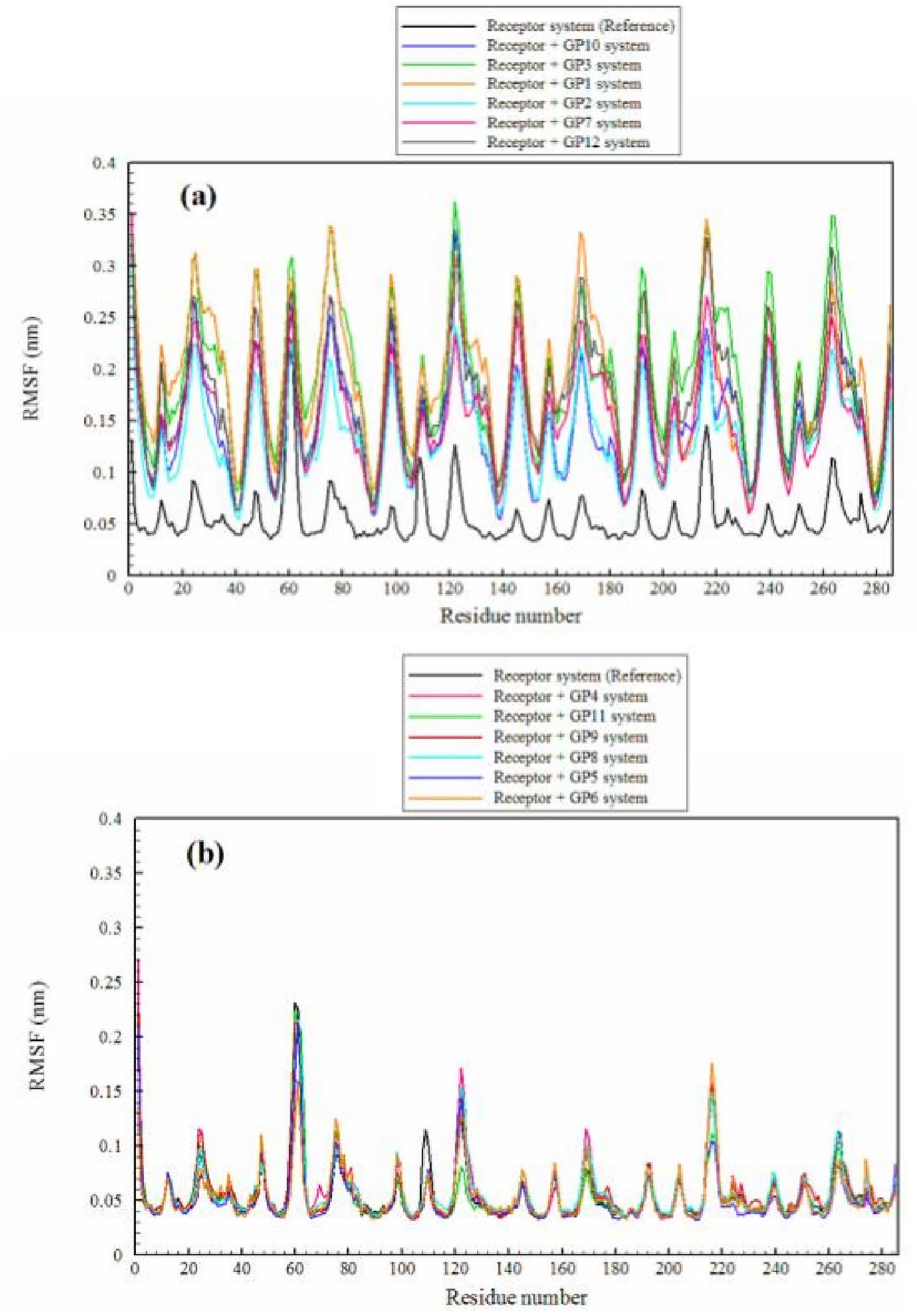
The RMSF of the receptor’s residues in different simulated systems. (**a**) Significant changes and high flexibility of the receptor; (**b**) no significant variations in the flexibility of the receptor.

RMSF analysis provides a better understanding of the dynamic behavior of proteins and offers insights into the structure–function relationships. This analysis can assist us in guiding experimental research and play a role in protein engineering strategies and drug discovery efforts. RMSF analysis can identify residues that experience significant fluctuations upon ligand binding. Amino acids with high RMSF values are generally associated with flexible or disordered regions of the protein. These regions may be involved in vital biological functions such as binding to other molecules or structural changes^58,59^. As shown in Fig. 7, the RMSF values for all receptor amino acids were evaluated in the presence of various ligands. As can be seen, there are two distinct behaviors in the presence of different ligands (ESI File, S3). The RMSF values for receptor amino acids in the presence of ligands **GP10**, **GP3**, **GP1**, **GP2**, **GP7,** and **GP12** undergo significant changes and demonstrate high flexibility, while they remain relatively rigid in the presence of ligands **GP4**, **GP11**, **GP9**, **GP8**, **GP5**, and **GP6** with no significant variations in the flexibility of receptor amino acids compared to the reference system (system consisting of the receptor only).

Ligand binding can induce structural changes in the protein, which affects the flexibility of amino acids. This phenomenon, known as induced fit, allows the protein to adjust its structure for optimal ligand binding^60,61^. To investigate the impact of flexibility on protein conformational changes, we focus on studying the free energy surface.

#### Free energy surface

**Figure 8:**
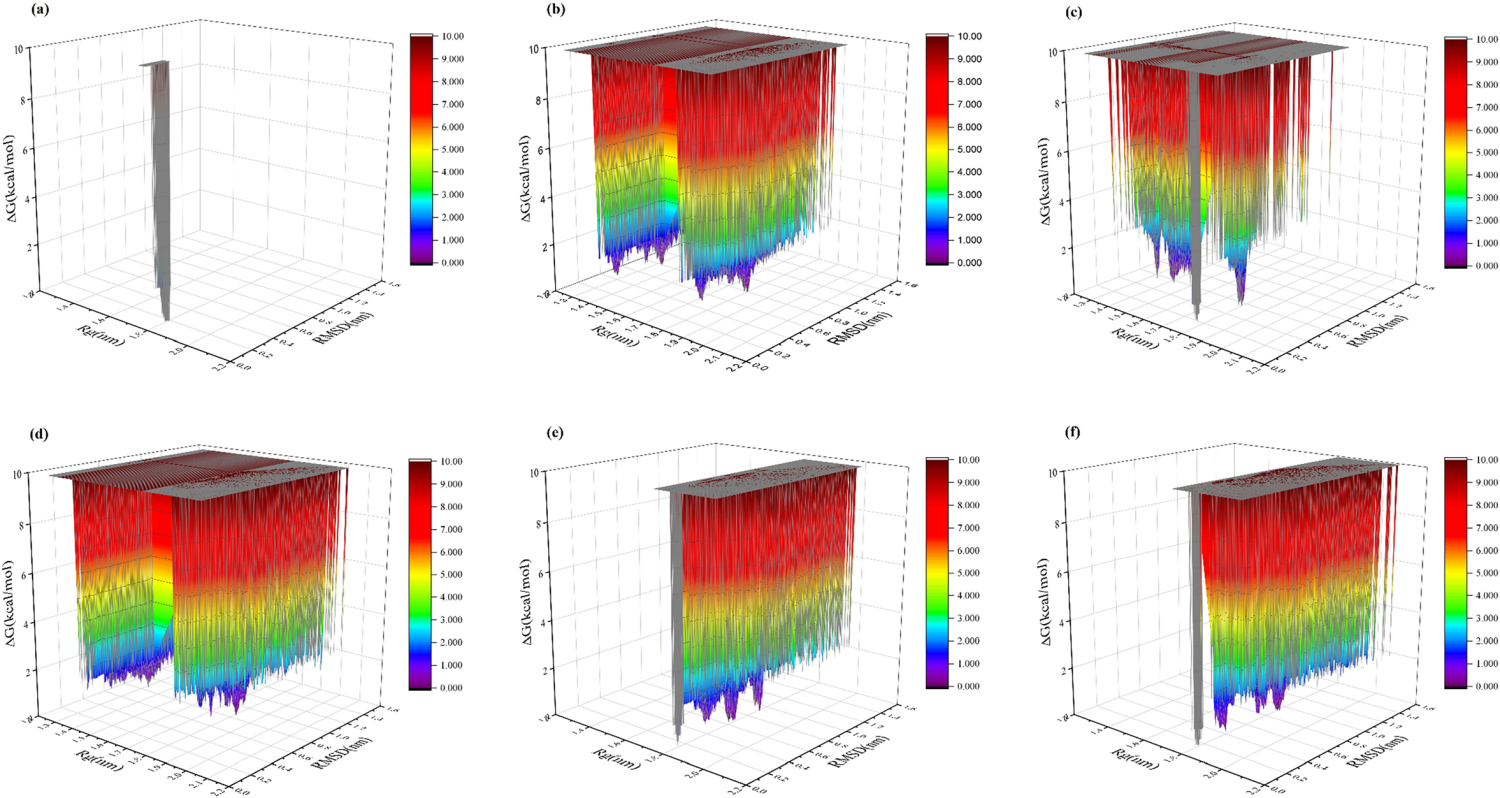
Free energy surface of the protein (receptor) in the presence of different ligands. (**a**) Reference system, (**b**) GP3, (**c**) GP5, (**d**) GP12, (**e**) GP4, (**f**) GP8.

Proteins in their natural environment exist not as single structures, but rather as a dynamic ensemble of configurations that are distributed over a range of energies and on a Free Energy Surface (FES) based on their probabilities of occurrence^62^. The FES provides insights into the structural dynamics of proteins, pathways of folding/unfolding, binding events, and other thermodynamic properties. They can also help identify stable states or meta-stable intermediates, determine transition states, and elucidate underlying mechanisms of protein function. The information obtained from FES is valuable in various fields including drug design, protein engineering, and understanding protein folding and structural changes^63^. In this study, we explored the free energy surface (FES) using two key collective variables-gyration radius (Rg) and root mean square deviation (RMSD)-to characterize the motions and coordinates of the target protein (receptor), as illustrated in Fig. 8 and Supplementary Information (ESI) File S2. It should be noted that a larger Rg indicates a more expanded structure, while a smaller Rg corresponds to a more compact or folded structure. Additionally, lower RMSD values indicate greater similarity between simulated structures and the reference, while higher RMSD values indicate greater structural deviation. The results obtained demonstrate that a structure with Rg and RMSD values of 1.8 nm and 0.2 nm, respectively, represents the most stable conformation of the protein (receptor) in the absence of a ligand (reference system). With the addition of ligands **GP3**, **GP6**, and **GP12**, the dominant observed configuration in the reference system completely disappears, and structures with different and diverse configurations with Rg equal to 1.3 nm and 1.8 nm, and RMSD equal to 0.5 nm and 0.8 nm in the presence of **GP3** emerge. Therefore, the structural similarity of the protein is completely lost. However, in the presence of **GP6**, the most stable protein configuration is also observed with Rg equal to 1.8 nm and RMSD equal to 0.6 nm and 0.8 nm, and there have been no significant changes in Rg in the presence of this ligand. With the addition of the ligand **GP12** to the protein, significant changes in Rg and RMSD are observed, such that structures with Rg equal to 1.3 nm and 1.8 nm, and RMSD equal to 0.8 nm are evident thermodynamically. It appears that the most significant structural changes in the protein are caused by the presence of these three ligands. Despite the influence of additional ligands, the original protein configuration remains discernible within the system, contributing thermodynamically to its stable configurations. The presence of the ligand **GP5** induces noticeable changes in the protein’s Rg, leading to a more compact conformation. The protein adopts stable configurations with an RMSD of approximately 0.8 nm. In the presence of ligands **GP9** and **GP8**, the protein experiences changes solely in RMSD, with no significant alterations in structural compactness observed. Additionally, the presence of ligands **GP4**, **GP10**, **GP1**, **GP2**, and **GP11** leads to the creation of stable configurations with partial changes in Rg and significant changes in RMSD. Finally, the least changes in protein configuration resulting from the presence of the ligand **GP7** are observed, such that it is only associated with an RMSD of 0.4 nm and no noticeable change in Rg.

#### Hydrogen bonding

Hydrogen bonds contribute to the stability and structural integrity of proteins. They form between the electronegative atoms of oxygen or nitrogen in the peptide backbone and hydrogen atoms connected to these atoms. These bonds help maintain secondary structures such as alpha helices and beta sheets. Proteins are not static molecules; they undergo structural changes to perform their functions. Hydrogen bonds are dynamic interactions that can form, break, and rearrange during these structural changes^64,65^. The findings reveal a slight increase in the number of intramolecular hydrogen bonds (protein/protein), as depicted in Table 3. This emphasizes the necessity for a more comprehensive investigation into the various intramolecular hydrogen bonds within the protein and their correlation with protein structure. As seen in Table 2, the reference system exhibits 118 anti-parallel bridges for the protein. With the addition of the ligand and its interaction with the protein, significant changes occur in the number of anti-parallel bridges. Specifically, the protein shows the most pronounced changes in the presence of **GP10**, **GP11**, **GP12**, **GP4**, and **GP5**, resulting in a significant reduction in the number of anti-parallel bridges (Table 3). It should be noted that anti-parallel bridges are typically found in the secondary structures of proteins, especially in β-sheets.

**Table 3:** Different types of hydrogen bonds (H-bond) between residues of the KEAP1 protein.

Type of H-bond system	Antiparallel Bridges	O(I) → H-N(I + 2)	O(I) → H-N(I + 3)	O(I) → H-N(I + 4)	O(I) → H-N(I + 5)	O(I) → H-N(I-1)	O(I) → H-N(I-2)	O(I) → H-N(I-3)	O(I) → H-N(I-5)	Between protein/protein
Receptor (Reference)	118	10	4	5	5	0	2	6	6	214.355
Recep + (**GP3**)	118	18	5	5	6	0	1	7	6	223.700
Recep + (**GP5**)	110	17	5	5	5	0	1	6	6	221.530
Recep + (**GP7**)	112	17	3	5	6	1	1	6	6	223.941
Recep + (**GP12**)	110	19	2	6	5	0	1	6	6	225.368
Recep + (**GP4**)	117	16	7	6	4	1	2	5	6	223.307
Recep + (**GP1**)	118	27	5	6	4	1	1	7	6	222.808
Recep + (**GP2**)	121	23	9	5	4	0	2	7	6	222.965
Recep + (**GP8**)	119	19	5	5	6	1	1	6	6	222.145
Recep + (**GP11**)	117	14	6	6	5	0	1	6	6	222.301
Recep + (**GP9**)	118	8	6	5	4	1	1	6	5	224.382
Recep + (**GP10**)	104	15	4	6	5	1	1	6	6	221.870
Recep + (**GP6**)	110	13	3	6	5	0	1	6	6	222.683

The β-sheets consist of multiple peptide chains connected by anti-parallel bridges. Hydrogen bonds formed between adjacent chains play a crucial role in maintaining the stability and integrity of β-sheets. The hydrogen bonds formed through anti-parallel bridges contribute to the structural stability and functional properties of proteins ^66–69^. Therefore, reducing their number can significantly impact protein instability and the loss of functional properties. Consequently, a severe decrease in hydrogen bonds formed through anti-parallel bridges can be highly significant in terms of structural and functional changes in proteins. Another significant change can be observed in the number of hydrogen bonds between O(I) → H-N(I + 2). This hydrogen bond is typically found in the regular secondary structures of proteins, such as α-helices and β-sheets. In an α-helix, the carbonyl oxygen of an amino acid residue at position ‘I’ forms a hydrogen bond with the amide hydrogen of the amino acid residue at position ‘I + 2’ (two positions ahead in the sequence). This hydrogen bond contributes to the stability and strength of the α-helix structure. In the reference system, 10 such bonds have been established, and a significant increase is observed in the presence of all ligands, except for **GP9**. In O(I) → H-N(I + 3), the hydrogen bond decreases in the presence of certain ligands (**GP4**, **GP12**, **GP11**), while it significantly increases in the presence of other ligands, further confirming the formation and enhanced stability of the α-helix structure in this protein. Finally, the hydrogen bond between position ‘I’ and the preceding positions is not very pronounced and does not lead to drastic changes.

To comprehensively study and evaluate the thermodynamic behavior of different antioxidant compounds binding to protein residues, we conducted an assessment of the thermodynamic favorability of these interactions using free energy calculations, as presented in Table S2^70^. In addition, considering that hydrogen bond formation in molecular dynamics simulations typically occurs within distances less than 3.5 Å^71–73^, we assessed the residues binding to each antioxidant by examining distances between their center of mass (COM) and nearby protein residues, as detailed in Fig. S3 and Table S3 (Supporting Information File).

## Conclusion

This research presents the experimental application of a machine learning model to design antioxidant peptides (AOPs) de novo. The optimized generative model was utilized to generate twelve novel sequences of AOPs. To identify the most promising peptides for synthesis, the generated peptides were ranked by DFT calculation based on their E_HOMO_ and Eg. The peptide **GP12**, with an E_HOMO_ of − 4.92 eV, emerged as a formidable electron donor, suggesting heightened antioxidant properties. Peptides **GP9**, **GP10**, and **GP12** with the highest E_HOMO_ along with three other randomly selected peptides were synthesized for their antioxidant capacity and anti-hemolytic activity. Three (**GP9**, **GP10**, and **GP12**) out of the six synthesized peptides showed antioxidants active to the extent of ascorbic acid with non-hemolytic properties. The RMSF and FES analysis of proteins in the presence of the computer-generated peptides **GP1–GP12** showed that different sequences induce significant structural changes in proteins, affecting their stability and flexibility. Additionally, the number of hydrogen bonds, especially anti-parallel bridges and those within secondary structures, varies with ligand presence, impacting protein stability and function.

From the above results and observations based on the MD simulations and the antioxidant assays, **GP12** shows the best results to be included for further analysis towards in vitro and in vivo assays for both Keap1 and antioxidant activity analysis. It can be claimed with certainty that the machine learning methods, along with DFT calculations and MD analysis, are applicable to automated peptide design in a prospective setting without having to extract, purify, synthesize, and test large sets of peptides. However, as the results show, the model is not capable of generating active AOPs for the hydroxyl scavenging test. This limitation arises from the fact that the currently available dataset lacks information regarding the specific testing methods used to assess the activity of each antioxidant. Therefore, the options for creating antioxidant peptides with specific activity towards any of the available mechanisms are constrained. Despite attempts to identify each antioxidant’s activities based on their reference papers, the variations in experimental methods, some of which are no longer popular in contemporary research, have led to ambiguities. Furthermore, most of the reported peptides have only one recorded activity, potentially leading to biased interpretations. To address these challenges and enhance the discovery of active AOPs, a high throughput screening of the current peptides in the dataset is necessary, utilizing specific and fixed antioxidant activity assays such as DPPH, hydroxyl, and ROS activity. This approach would not only enrich the dataset but also offer a comprehensive understanding of the interrelation of amino acids in the sequence.

In order to advance the methods and accelerate the discovery of AOPs using machine learning and quantum calculations, several suggestions is proposed:

1. Exploration of additional deep learning layers such as convolutional layers, LSTM layers, simple RNN layers, and Attention-based layers to analyze the model’s performance^74,75^.
2. Development of alternative architectures including Autoencoders, VAEs, and GPT-based generative models^76–78^. Autoencoder models will give a chance to develop and benchmark classification models for antioxidant activities besides their potential to be a useful generative model while the GPT’s embedding layer can also be used for classification, besides the possibility of using fine-tuned BERT-based models for the classification tasks^79–82^.
3. Integration of reinforcement learning to develop AOPs with dual activity towards the KEAP1 protein and antioxidant activity. This could involve the use of a classification model for AOP prediction and a molecular docking approach for the KEAP1 protein to train the model to generate active peptides targeting both parameters. Also, classification or regression models for KEAP1 can also be created. However, the limitation based on the number of peptides in the dataset could create challenges to developing models sensitive towards peptide sequences and their representation.
4. Furthermore, the exploration of unnatural amino acid-based AOPs presents a promising avenue, considering their potential to overcome the limitations of natural peptides in the human body. To enable this exploration, alternative representations such as molecular fingerprints for these peptides could be considered for developing machine learning models. However, as this chemical space is not well understood and explored, developing machine learning models to predict or generate new data points is not very suited as we do not have enough data to train a generative model or to be sure of a capable classification model that could distinguish the activity of a chosen sequence with natural and unnatural amino acids. With future advancements and explorations on the mentioned topics, the field of designing AOPs is poised to make a significant step in the future.
5. While our DFT calculations, which focus on HOMO and LUMO energies, have successfully identified promising antioxidant peptides, it is crucial to recognize the inherent generalization of this approach. For instance, when comparing the possible activity of the selected peptides, based on their calculated parameters and the experimental results, we can clearly see a challenge towards the accuracy of this general strategy. This strategy has been done and selected as a simple method to identify active or inactive AOPs. Our strategy towards selecting the final peptides for the synthetic and experimental validation phase was good based on the DPPH assay. However, it’s worth noting that **GP12** wasn’t the most active peptide against this assay. This information shows that besides the good accuracy towards the DPPH, those implementations are not enough to predict and rank the chosen peptides perfectly. Additionally, the proposed ranking based on the calculated properties did not fully align with the final ranking observed in the experimental method. This strategy also did not show a very reliable method towards the hydroxyl assay. To enhance the predictive power of our findings and ensure robust conclusions, future research endeavors should engage in a thorough examination of all potential antioxidant mechanisms. The incorporation of DFT methods alongside transition state analysis could provide valuable insights for more efficient peptide design. In summary, while our DFT calculations offer novel insights, a comprehensive exploration of antioxidant mechanisms is indispensable for advancing the field of peptide-based antioxidants.

## Supplementary Information

Supplementary Information.

## Data Availability

The datasets generated and/or analysed during the current study are available in the https://github.com/mephisto121/DeepGenAntiOxidantPeptide.
