# Author Correction: De novo antioxidant peptide design via machine learning and DFT studies

**DOI:** 10.1038/s41598-026-55147-y

**Published:** 2026-06-04

**Authors:** Parsa Hesamzadeh, Abdolvahab Seif, Kazem Mahmoudzadeh, Mokhtar Ganjali Koli, Amrollah Mostafazadeh, Kosar Nayeri, Zohreh Mirjafary, Hamid Saeidian

**Affiliations:** 1 Department of Chemistry, Science and Research Branch, Islamic Azad University, Tehran, Iran; 2 Dipartimento di Fisica, Universita’ di Padova, Via Marzolo 8, 35131 Padua, Italy; 3 Department of Chemistry, University of Turin, Via Pietro Giuria 7, 10125 Turin, Italy; 4 Department of Organic Chemistry and Oil, Faculty of Chemistry, Shahid Beheshti University, Tehran, Iran; 5 Department of Chemistry, University of Kurdistan, Sanandaj, Iran; 6 Cellular and Molecular Biology Research Center, Health Research Institute, Babol University of Medical Sciences, Babol, Iran; 7 Student Research Committee, Babol University of Medical Sciences, Babol, Iran; 8 Department of Science, Payame Noor University (PNU), PO Box: 19395-4697, Tehran, Iran

Correction to: *Scientific Reports* 10.1038/s41598-024-57247-z, published online 18 March 2024

The original Article contained errors where the DPPH assay was described incorrectly in the Materials and methods section under the subheading ‘DPPH scavenging activity assay’. Therefore, the sentence:

“The peptide sample (250 μL) with DPPH solution (250 μL) was mixed and stirred in the dark for 30 min at room temperature.”

Now reads:

“The peptide sample (250 μL) with DPPH solution (250 μL) in ethanol (1.0 mL) was mixed and stirred in the dark for 30 min at room temperature.”

In addition, a sentence in the legend of Figure 5 was omitted. Therefore, the legend:

“**Figure 5.** (**a**) Comparison of the color intensity of different samples based on the antioxidant activity in DPPH assay. (**b**) Comparison of the color intensity of different samples based on the antioxidant activity in the hydroxyl radical scavenging activity assay. It should be mentioned that ascorbic acid was used as the positive control (C+) and methanol was used as the negative control (C−). (**c**) The DPPH radical scavenging activity of the six synthesized peptides compared to ascorbic acid (Asc) as a positive control. (**d**) The hydroxyl radical scavenging activity of the synthesized peptides compared to the Asc as a positive control.”

Now reads:

“**Figure 5.** (**a**) Comparison of the color intensity of different samples based on the antioxidant activity in DPPH assay. (**b**) Comparison of the color intensity of different samples based on the antioxidant activity in the hydroxyl radical scavenging activity assay. It should be mentioned that ascorbic acid was used as the positive control (C+) and methanol was used as the negative control (C−). (**c**) The DPPH radical scavenging activity of the six synthesized peptides compared to ascorbic acid (Asc) as a positive control. (**d**) The hydroxyl radical scavenging activity of the synthesized peptides compared to the Asc as a positive control. It should be noted that the concentrations provided refer to those of the stock solution utilized, rather than the final concentrations observed in the assays.”

The original Article has been corrected.

