# Supplementary material for: De novo antioxidant peptide design via machine learning and DFT studies
Source: Sci Rep. 2024 Mar 18;14:6473. doi: 10.1038/s41598-024-57247-z (PMC10948870; doi:10.1038/s41598-024-57247-z)
Supplement: Supplementary file 1 — Supplementary Information. [file 41598_2024_57247_MOESM1_ESM.docx]

**Electronic Supplementary Information (ESI) for**

**Figure S1:** The RMSF of the receptor's residues in different simulated systems.

***De Novo* Antioxidant Peptide Design *via* Machine Learning and DFT studies**

Parsa Hesamzadeh^1^, Abdolvahab Seif ^2,3^, Kazem Mahmoudzadeh^3^, Mokhtar Ganjali Koli^4^, Amrollah Mostafazadeh^6^, Kosar Nayeri^7^, Zohreh Mirjafary^1^, Hamid Saeidian^*8^

*^1^ Department of Chemistry, Science and Research Branch, Islamic Azad University, Tehran, Iran*

*^2^ Dipartimento di Fisica, Universita' di Padova, Via Marzolo 8, 35131, Padova, Italy*

***^3^****Department of Chemistry, University of Turin, Via Pietro Giuria 7, 10125, Torino, Italy*

*^4^ Department of Organic Chemistry and Oil, Faculty of Chemistry, Shahid Beheshti University, Tehran, Iran*

*^5^Department of Chemistry, University of Kurdistan, Sanandaj, Iran*

*^6^Cellular and Molecular Biology Research Center, Health Research Institute, Babol University of Medical Sciences, Babol, Iran*

*^7^Student Research Committee, Babol University of Medical Sciences, Babol, Iran*

*^8^Department of Science, Payame Noor University (PNU), PO Box: 19395-4697, Tehran, Iran*

*E-Mail:* [*Saeidian1980@gmail.com*](mailto:Saeidian1980@gmail.com)

**Content**

[**S1 Machine Learning 2**](#_gjdgxs)

[S1.1 Deep Generative Model 2](#_30j0zll)

[S1.1.1 Pre-trained Generative Model Details 2](#_1fob9te)

[S1.1.2 Fine-tuned AOP Generative Model Details 2](#_3znysh7)

[S1.2 Antioxidant Classification Model 3](#_2et92p0)

[S1.2.1 Classification Model details 3](#_tyjcwt)

[S1.2.2 Performance Evaluation 4](#_3dy6vkm)

[S1.3 Evaluation metrics 5](#_1t3h5sf)

[S1.3.1 ROC AUC Score (Receiver Operating Characteristic Area Under the Curve) 5](#_4d34og8)

[S1.3.2 Accuracy 6](#_2s8eyo1)

[S1.3.3 Precision 6](#_17dp8vu)

[S1.3.4 Matthews correlation coefficient 7](#_t2urqz43w1r8)

[**S2 Hemolysis Assay 8**](#_3rdcrjn)

[**S3 MD-simulations 9**](#_26in1rg)

# S1 Machine Learning

## S1.1 Deep Generative Model

### S1.1.1 Pre-trained Generative Model Details

*x = tf.keras.Input(shape=(19,))*

*x2 = tf.keras.layers.Embedding(*

*input_dim=vocab_size, output_dim=embedding_dim, mask_zero=True)(x)*

*h1 = layers.GRU(rnn_units, return_sequences=True, stateful=False)(x2)*

*h2 = layers.GRU(rnn_units, return_sequences=True, stateful=False)(h1)*

*yhat = tf.keras.layers.Dense(vocab_size)(h2)*

*train_model = tf.keras.Model(inputs=x, outputs=yhat)*

Epochs = 250, Batch_size = 64, Loss Function = Sparse Categorical Cross Entropy, Optimizer = Adam, Learning Rate = 0.001, Learning Rate Schduler = (**ReduceLROnPlateau** monitoring the training loss, factor=0.5, patience=5, min_lr=0.000001, verbose=1, min_delta=1e-5), Model Checkpoint = Based on training loss

### S1.1.2 Fine-tuned AOP Generative Model Details

*x = tf.keras.Input(shape=(19,))*

*x2 = tf.keras.layers.Embedding(*

*input_dim=vocab_size, output_dim=embedding_dim, mask_zero=True)(x)*

*h1 = layers.GRU(rnn_units, return_sequences=True, stateful=False)(x2)*

*h2 = layers.GRU(rnn_units, return_sequences=True, stateful=False)(h1)*

*yhat = tf.keras.layers.Dense(vocab_size)(h2)*

*Generateing_train_model = tf.keras.Model(inputs=x, outputs=yhat)*

*Generateing_train_model.load_weights("../input/gru-base-weights/checkpoint")*

*for i in range(3):*

*Generateing_train_model.layers[i].trainable = False*

*Generateing_train_model.summary()*

Epochs = 10k, Batch_size = 64, Loss Function = Sparse Categorical Cross Entropy, Optimizer = Adam, Learning Rate Schduler = (**ReduceLROnPlateau** monitoring the validation loss, factor=0.5, patience=5, min_lr=0.000001, verbose=1, min_delta=1e-5), Model Checkpoint = Based on validation loss

## S1.2 Antioxidant Classification Model

### S1.2.1 Classification Model details

*def model():*

*tf.random.set_seed(42)*

*input = layers.Input(shape=(1,), dtype="string")*

*vect = text_vectorizer(input)*

*x1 = layers.Embedding(22, 256)(vect)*

*x2 = layers.Conv1D(128,3)(x1)*

*x3 = layers.GlobalMaxPooling1D()(x2)*

*layer1 = tf.keras.layers.LayerNormalization(axis=-1)*

*xx = layer1(x3)*

*yhat = layers.Dense(1, activation = "sigmoid")(xx)*

*classification_model = Model(inputs = input, outputs = yhat)*

*return classification_model*

Epochs = 80, Batch_size = 10, Loss Function = Binary Cross Entropy, Number of Folds = 5, Optimizer = Adam, Learning Rate = 0.0003, Learning Rate Decay = 0.001, Learning Rate Schduler = (**ReduceLROnPlateau** monitoring the AUC-ROC score of validation data, factor=0.5, patience=5, min_lr=0.000001, verbose=1, min_delta=1e-5), Model Checkpoint = Based on validation data’s accuracy

### S1.2.2 Performance Evaluation

**Tabel S1** Performance Comparison of Models with 5-Fold Cross Validation

| **Model Name** | **Accuracy%** | **AUC-ROC%** | **Precision%** |
| --- | --- | --- | --- |
| Model_1 | 78.9 | 86.7 | 78.2 |
| Model_2 | 76.7 | 80.2 | 74.2 |
| Model_3 | 75.3 | 82.3 | 79.4 |
| Model_4 | 73.7 | 82.3 | 77.7 |
| Model_5 | 76.2 | 81.1 | 72.4 |
| **Average** | **76.18** | **82.5** | **76.4** |

## S1.3 Evaluation metrics

### S1.3.1 ROC AUC Score (Receiver Operating Characteristic Area Under the Curve)

The ROC AUC score is a performance metric for binary classification problems at various threshold settings. It measures the area under the ROC curve, which is a graphical representation of the true positive rate against the false positive rate. The ROC AUC score ranges from 0 to 1, where a higher score indicates better model performance. Sensitivity stands for true positive rate or recall and measures the proportion of actual positive instances that are correctly predicted by the model. It is a crucial metric in scenarios where the identification of positives is of higher importance. Specificity measures the proportion of actual negative instances that are correctly predicted by the model. It is important in situations where the focus is on correctly identifying negative cases.

**Formula:**

**
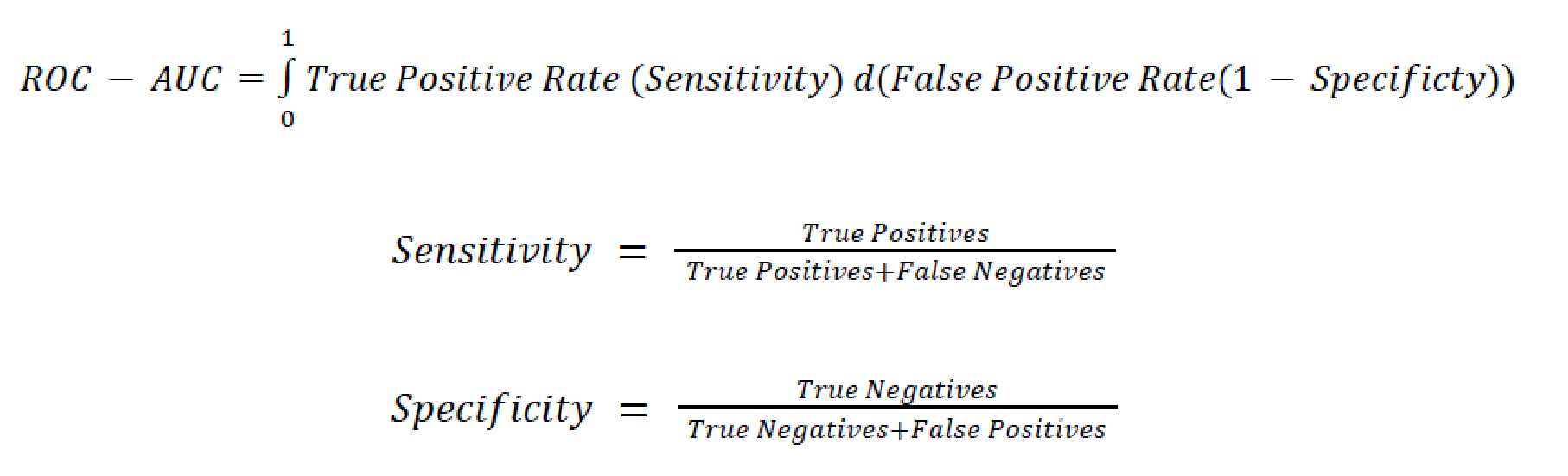
**

### S1.3.2 Accuracy

Accuracy is one of the most straightforward metrics, representing the ratio of correctly predicted instances to the total instances. It's a good measure for balanced datasets but might be misleading for imbalanced ones.

**Formula:**


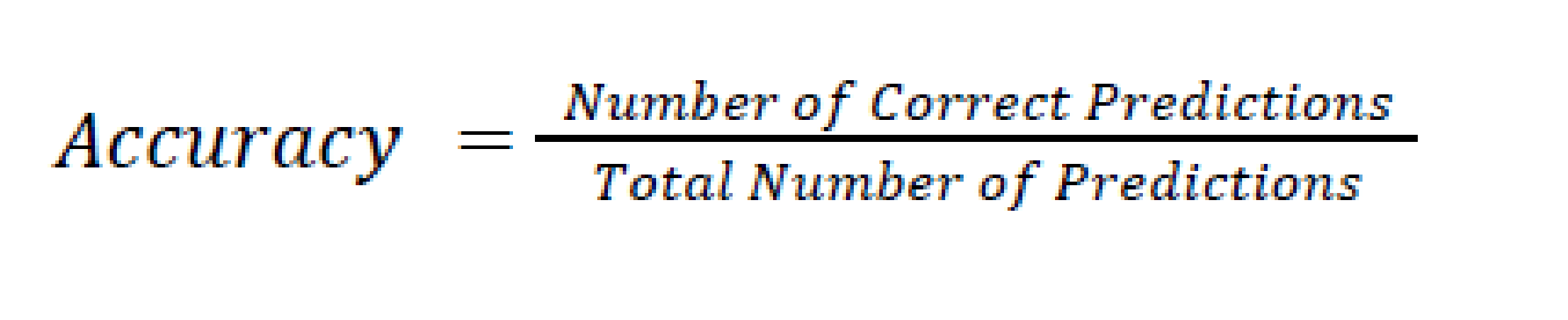


### S1.3.3 Precision

Precision is a metric that assesses the accuracy of the positive predictions made by a model. It's particularly useful when the cost of false positives is high. Precision is calculated as the ratio of true positive predictions to the sum of true positives and false positives.

**Formula:**


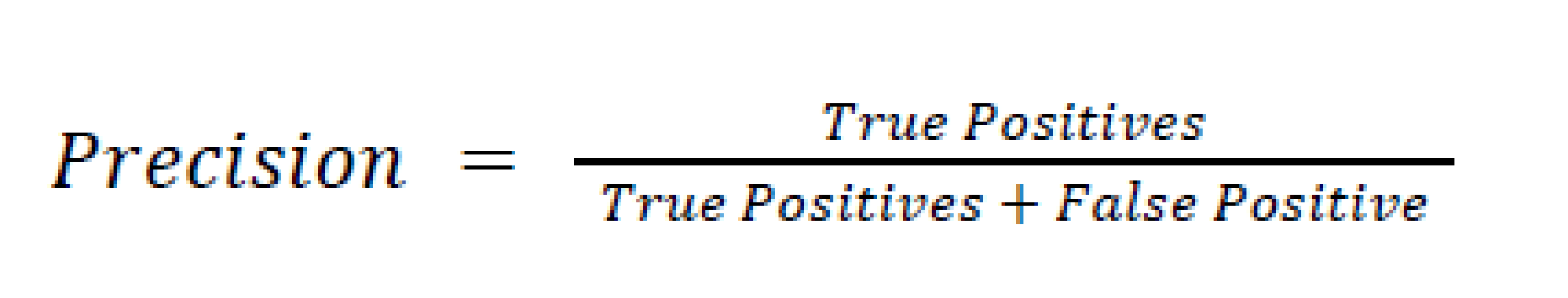


These metrics provide different perspectives on the performance of a classification model. ROC AUC evaluates the trade-off between sensitivity and specificity, accuracy provides an overall measure of correct predictions, and precision focuses on the accuracy of positive predictions.

### S1.3.4 Matthews correlation coefficient

The Matthews Correlation Coefficient (MCC) is a measure used in machine learning to evaluate the quality of binary classifications, particularly when dealing with imbalanced datasets. It takes into account true positives (TP), true negatives (TN), false positives (FP), and false negatives (FN) to provide a balanced assessment of the classifier's performance.

**Formula:**

$$\boldsymbol{MCC =}\frac{\boldsymbol{TP \times TN - FP \times FN}}{\sqrt{\boldsymbol{(TP+FP)(TP+FN)(TN+FP)(TN+FN)}}}$$

# S2 Hemolysis Assay

| 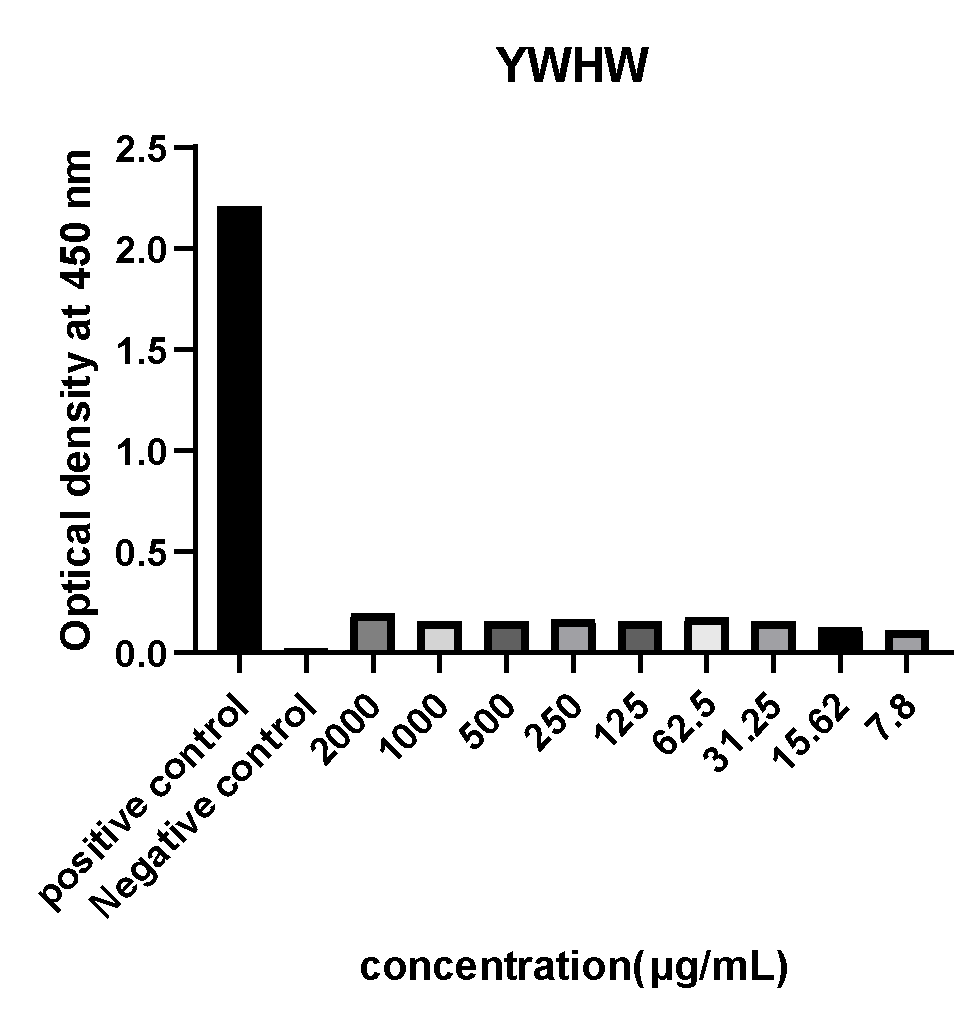 | 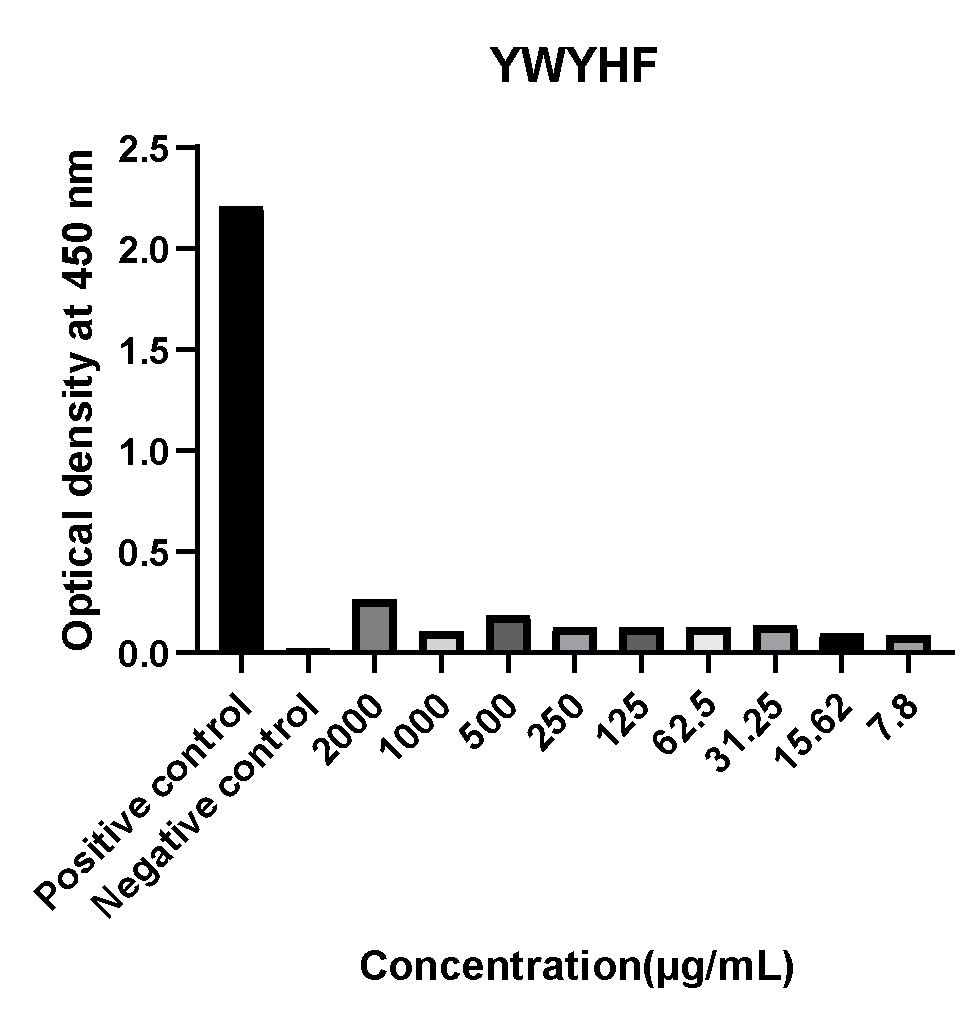 |
| --- | --- |
| 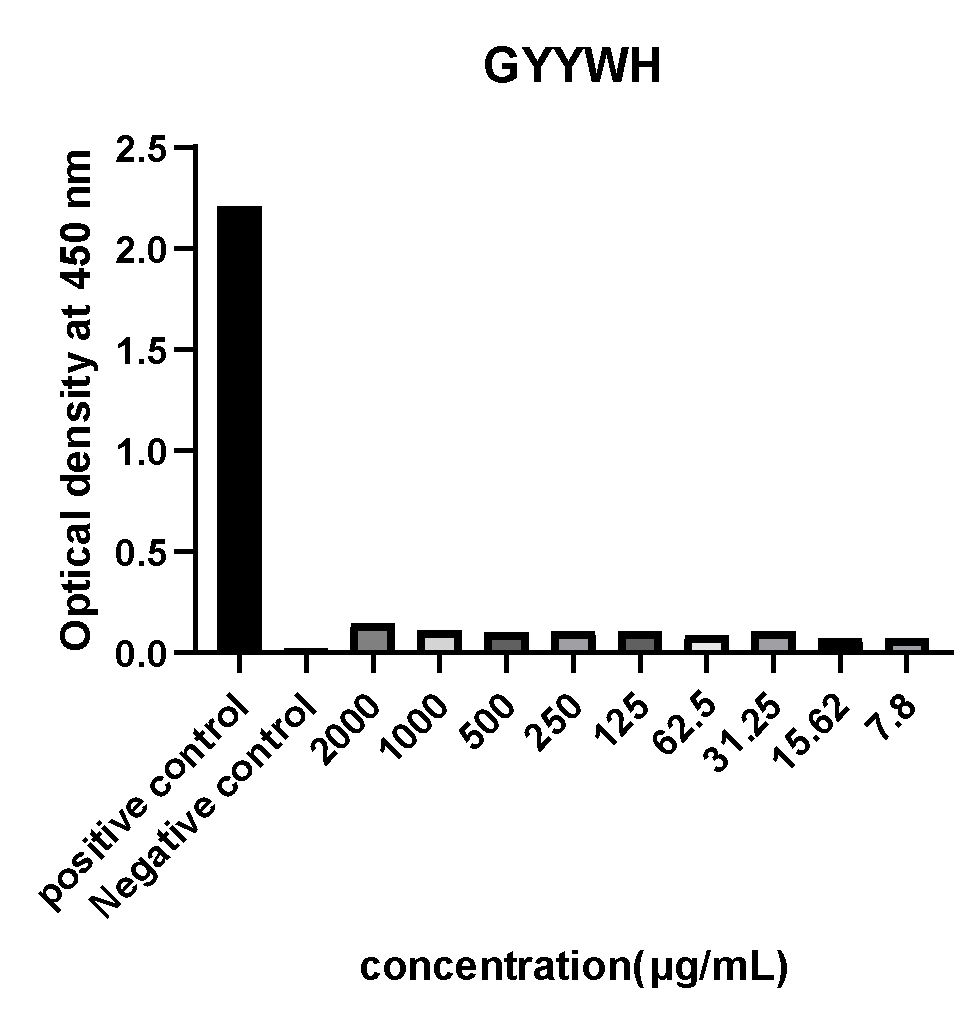 | 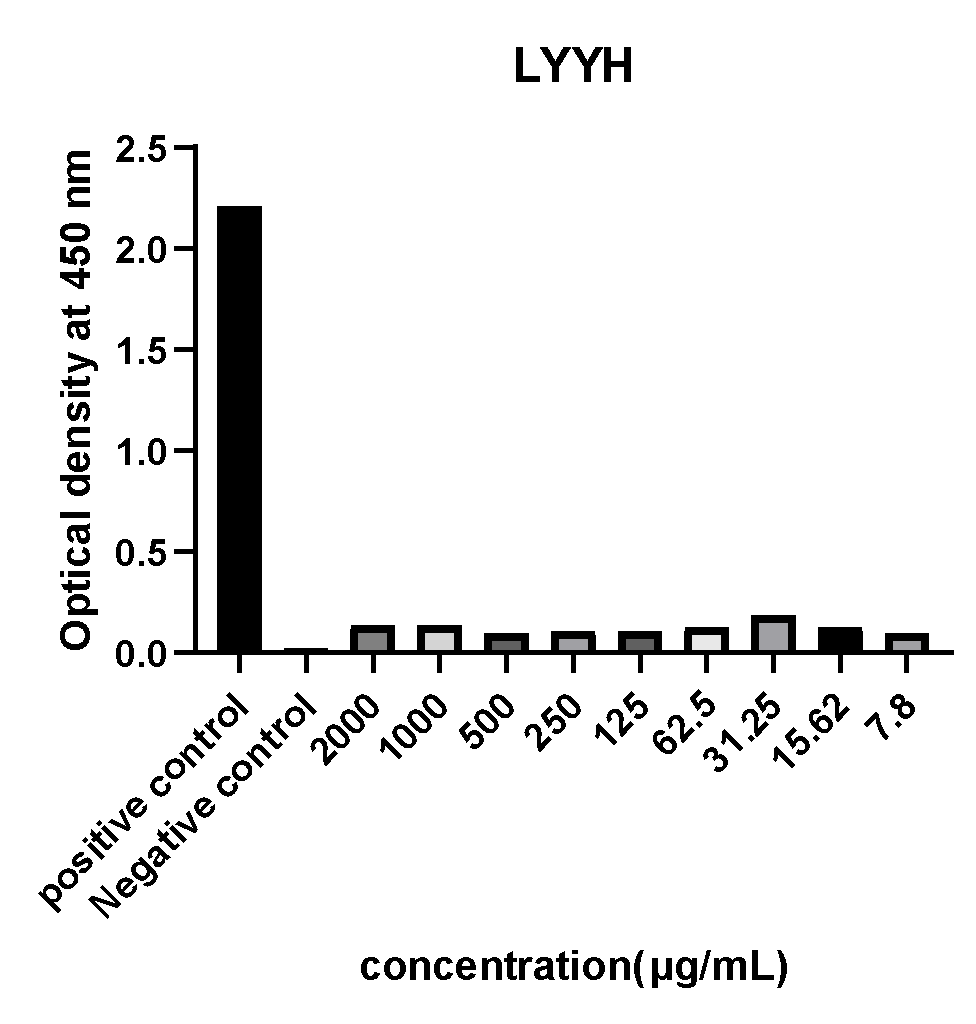 |
| 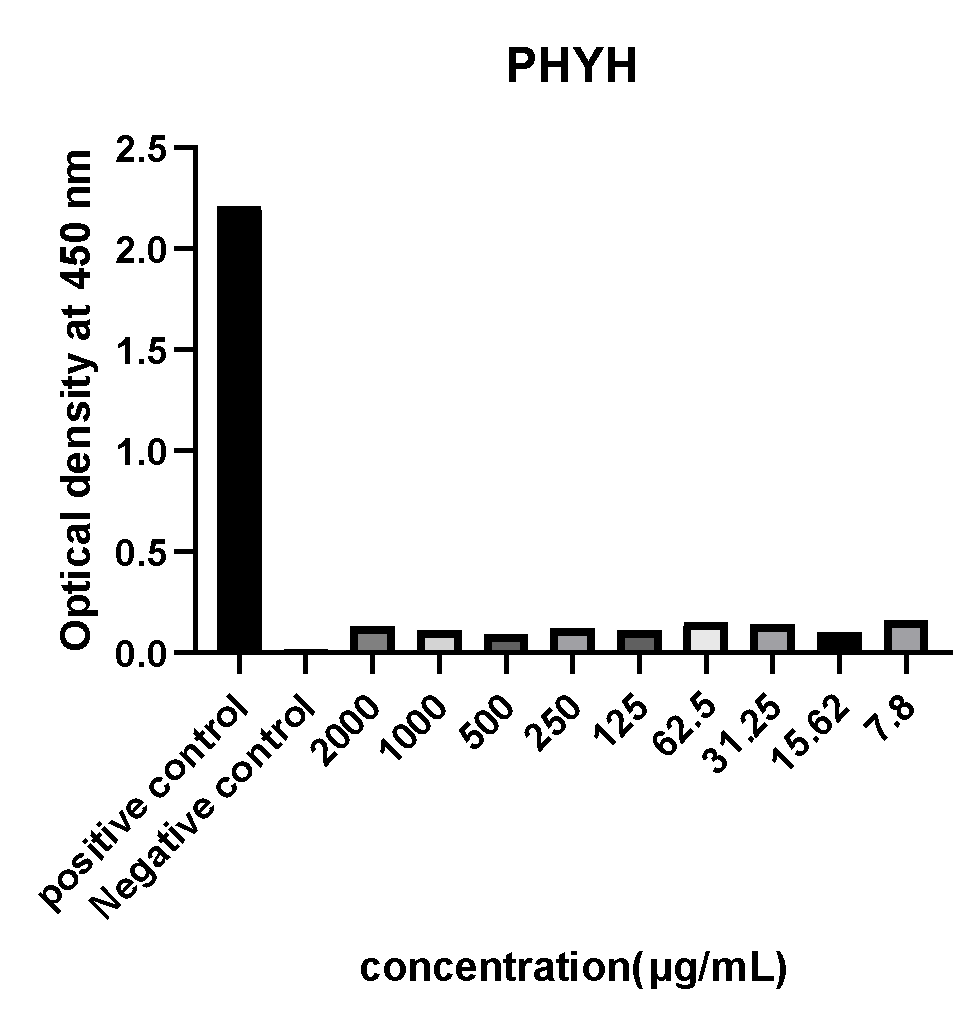 | 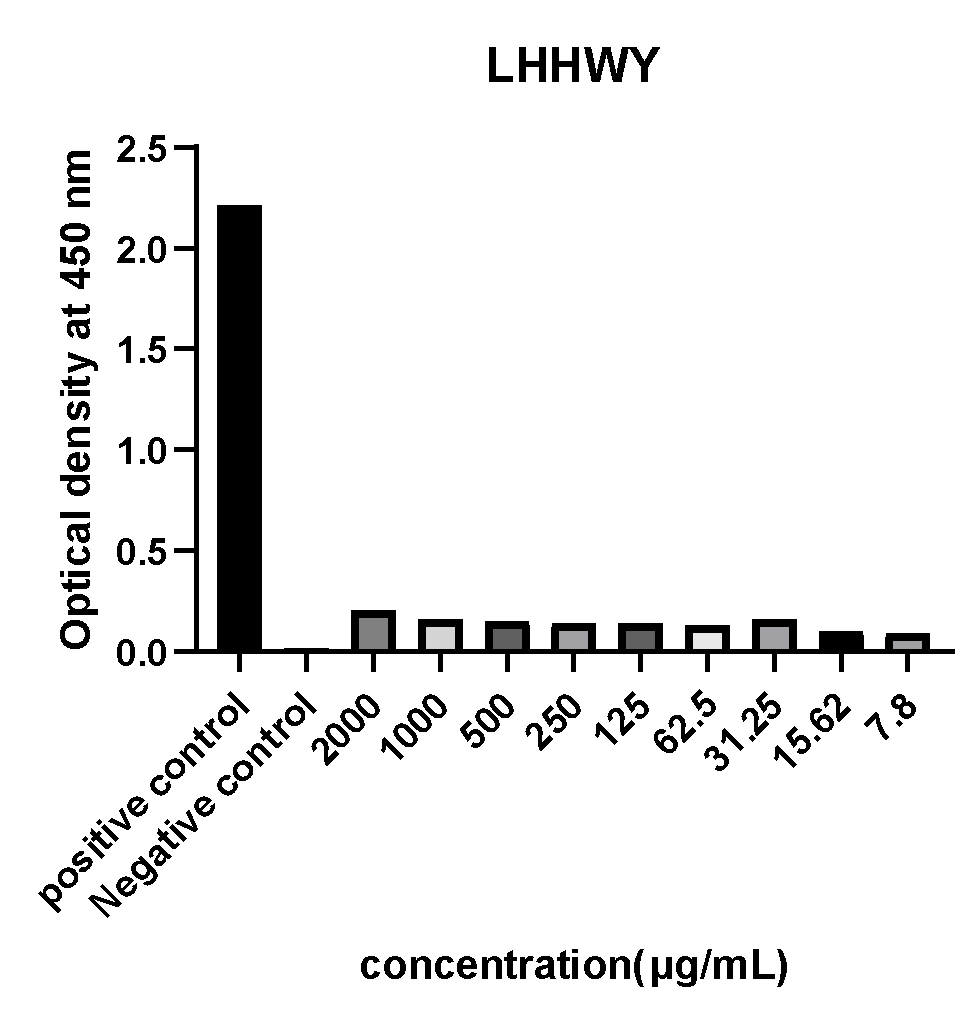 |

**Fig. S1** OD value for each tested peptide separately.

# S3 MD-simulations

**
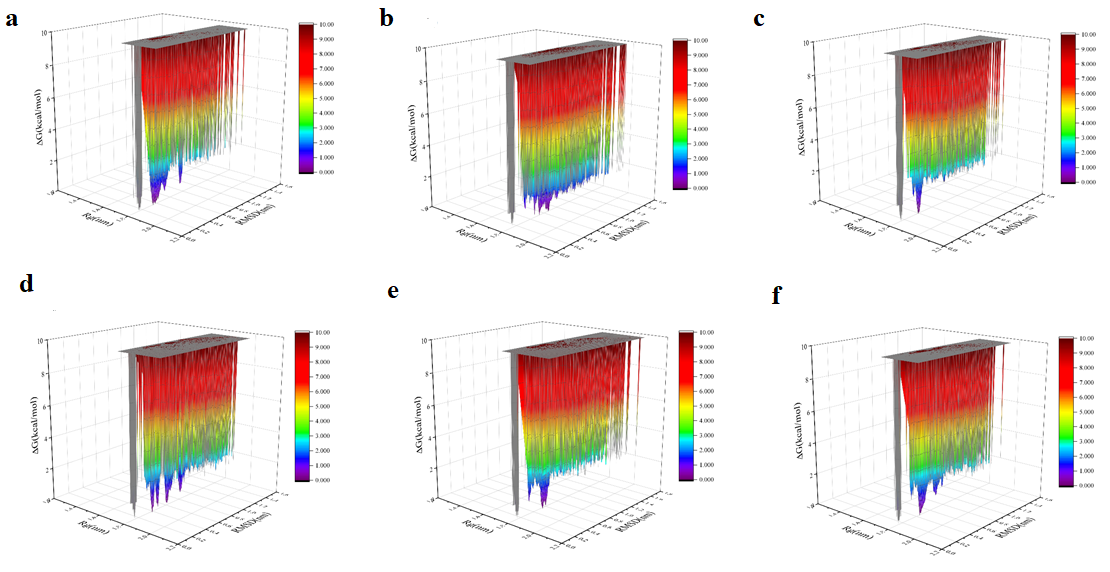
**

**
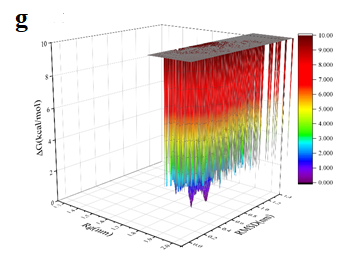
**

**Fig. S2** Free energy surface of the protein (receptor) in the presence of different ligands. a) **GP1**, b) **GP2**, c) **GP7**, d) **GP9**, e) **GP10**, f) **GP11**, g) **GP6**

**Binding Free Energy**

In order to quantify the difference in the binding affinity of complexes antioxidant/protein, their binding free energy was calculated using the Molecular Mechanics-Poisson Boltzmann Surface Area (MM-PBSA) approach. MM-PBSA is a fully atomistic method for the calculation of binding free energies that combines a molecular mechanics description of the protein complex with a continuous solvent model. It is widely used to evaluate interaction energies between proteins and biomolecules [1]. The trajectory ﬁles obtained from MD simulations were considered for calculation of binding free energy and the stability of the drug molecules in each selected target [2–4].

**Table S2.** Binding Free Energy of different antioxidant to KEAP1.

| Systems | Binging Free Energy (kJ/mol) |
| --- | --- |
| Receptor + **GP1** | -2.79 |
| Receptor + **GP2** | -4.23 |
| Receptor + **GP3** | -3.91 |
| Receptor + **GP4** | -7.34 |
| Receptor + **GP5** | -16.98 |
| Receptor + **GP6** | -22.66 |
| Receptor + **GP7** | -3.03 |
| Receptor + **GP8** | -9.21 |
| Receptor + **GP9** | -30.51 |
| Receptor + **GP10** | -8.33 |
| Receptor + **GP11** | -29.13 |
| Receptor + **GP12** | -8.24 |


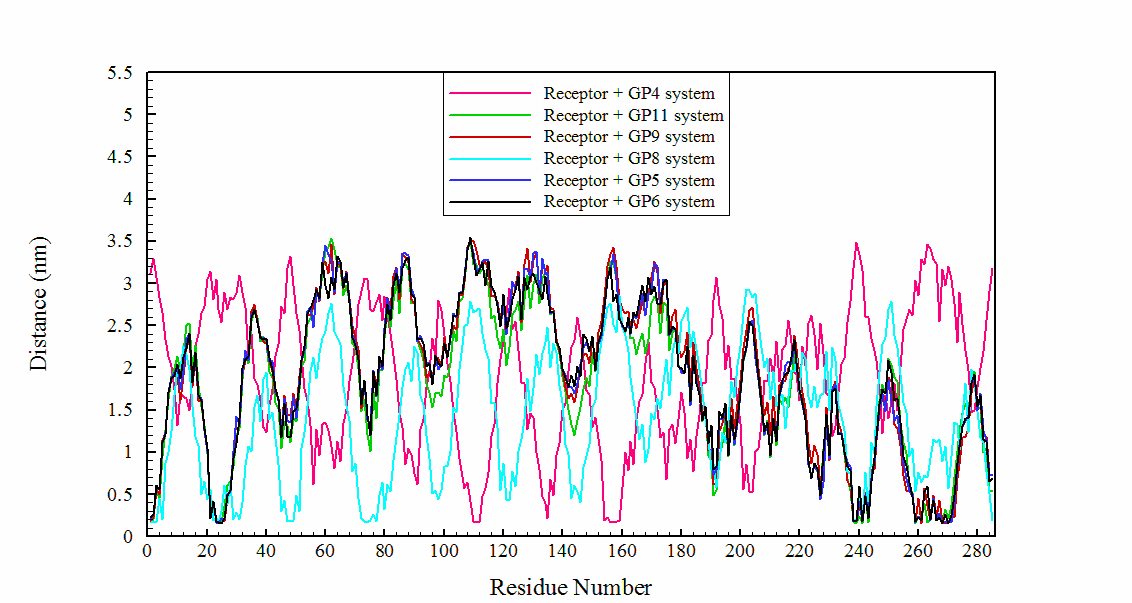

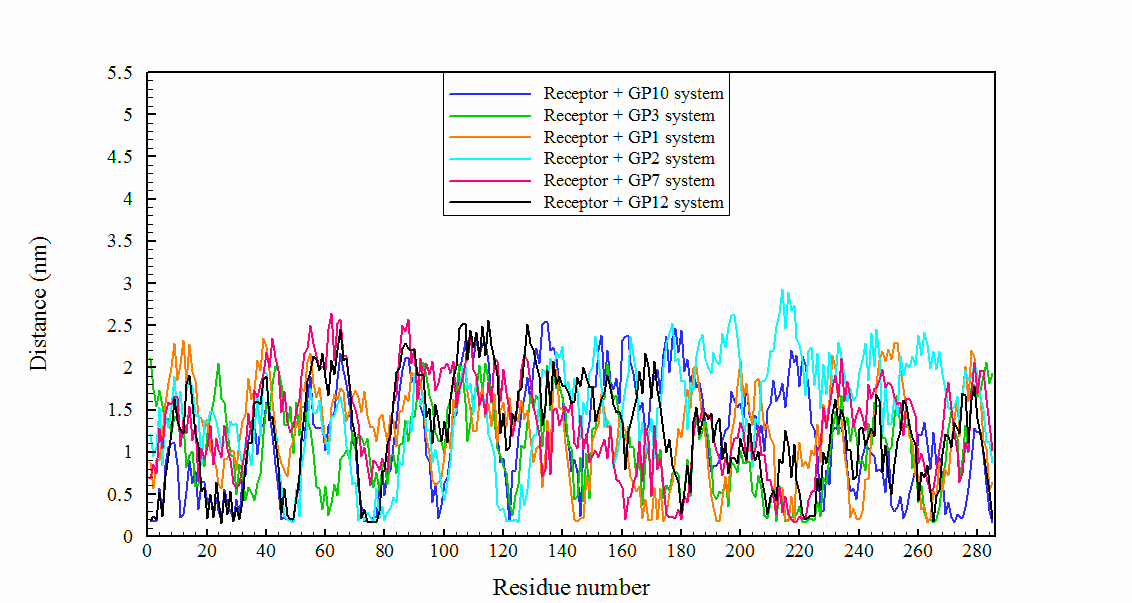


**Fig. S3** The distance of antioxidants from different residues of the KEAP1.

**Table S3.** The number and type of residues located within a distance of less than 3.5 Å from each antioxidant.

| Antioxidant | Number of residues | Type of residues |
| --- | --- | --- |
| **GP1** | 144, 145, 146, 147, 167, 169, 170, 172, 174, 175, 191, 192, 193, 212, 215, 216, 218, 237, 238, 239, 240, 241, 261, 263, 264 | LEU, ASN, ARG, LEU, TYR, GLU, ARG, GLU, ARG, MET, LEU, HIS, ASN, ARG, VAL, GLU, GLU, VAL, HIS, GLN, GLY, ARG, ASP, ASP, THR |
| **GP2** | 46, 47, 48, 49, 51, 71, 72, 73, 74, 75, 76, 78, 80, 81, 82, 119, 120, 121, 122, 123, 124, 125, | VAL, GLY, GLY, LEU, TYR, CYS, TYR, ASN, PRO, MET, THR, GLN, SER, PRO, CYS, TYR, GLU, PRO, GLU, ARG, ASP, GLU |
| **GP3** | 59, 61, 123, 180, 208, 209, 212, 215, 216, 217, 218, 219, 221, 222, 223, 224, 225, 227, 262, 263, 264, 265, 266, 267 | SER, ASP, ARG, ASN, ASN, SER, ARG, VAL, GLU, THR, GLU, THR, THR, PHE, VAL, ALA, PRO, LYS, PRO, ASP, THR, ASP, THR, TRP |
| **GP4** | 110, 111, 112, 135, 154, 155, 156, 157, 158, 159 | CYS, ILE, HIS, ARG, PHE, ASP, GLY, THR, ASN, ARG |
| **GP5** | 1, 2, 21, 23, 24, 25, 26, 238, 239, 241, 243, 259, 260, 261, 264, 266, 267, 268, 269, 270, 271 | GLY, ARG, TYR, ASN, PRO, SER, ASP, GLY, HIS, GLN, ARG, TYR, CYS, TYR, ASP, THR, THR, TRP, SER, GLU, VAL, THR |
| **GP6** | 1, 2, 21, 23, 24, 25, 26, 238, 239, 241, 243, 259, 260, 261, 264, 266, 267, 268, 269, 270, 271 | GLY, ARG, TYR, ASN, PRO, SER, ASP, GLY, HIS, GLN, ARG, TYR, CYS, TYR, ASP, THR, THR, TRP, SER, GLU, VAL, THR |
| **GP7** | 161, 175, 176, 177, 178, 179, 180, 212, 214, 216, 217, 218, 219, 220, 221, 222 | ASN, MET, ILE, THR, ALA, MET, ASN, ARG, ASP, GLU, THR, GLU, THR, TRP, THR |
| **GP8** | 1, 2, 3, 5, 22, 23, 24, 25, 29, 30, 31, 45, 47, 48, 49, 51, 72, 73, 74, 75, 76, 77, 80, 238, 239, 241, 243, 285 | GLY, ARG, LEU, TYR, ASN, PRO, SER, ASP, LEU, ARG, LEU, VAL, GLY, GLY, LEU, TYR, TYR, ASN, PRO, MET, THR, ASN, SER, HIS, GLN, ARG, TYR, THR |
| **GP9** | 1, 2, 21, 23, 24, 25, 26, 238, 239, 241, 243, 259, 261, 264, 266, 268, 269, 270, 271, 272 | GLY, ARG, TYR, PRO, SER, ASP, GLY, HIS, GLN, ARG, TYR, CYS, ASP, THR, THR, SER, GLU, VAL, THR, ARG |
| **GP10** | 1, 2, 3, 5, 11, 12, 17, 22, 24, 25, 28, 29, 31, 45, 46, 47, 48, 49, 73, 74, 75, 76, 77, 98, 122, 146, 227, 228, 252, 253, 255, 256, 269, 271, 272, 273, 274, 275, 276, 284, 285 | GLY, ARG, LEU, TYR, PHE, ARG, TYR, ASN, SER, ASP, TRP, LEU, LEU, VAL, VAL, GLY, GLY, LEU, ASN, PRO, MET, THR, ASN, ASP, GLU, ARG, LYS, HIS, THR, PHE, ASP, SER, GLU, THR, ARG, MET, THR, SER, GLY, VAL, THR |
| **GP11** | 1, 2, 21, 22, 23, 24, 25, 238, 239, 240, 241, 243, 259, 260, 261, 263, 264, 266, 267, 268, 269, 270, | GLY, ARG, TYR, ASN, PRO, SER, ASP, HIS, GLN, , GLY, ARG, TYR, CYS, TYR, ASP, ASP, THR, THR, TRP, SER, GLU, VAL |
| **GP12** | 1, 2, 3, 5, 20, 22, 25, 27, 29, 30, 31, 45, 47, 48, 49, 72, 73, 74, 75, 76, 77, 78, 180, 209, 221, 222, 223, 224, 225, 265, 285 | GLY, ARG, LEU, TYR, ALA, ASN, ASP, THR, LEU, ARG, LEU, VAL, GLY, GLY, LEU, TYR, ASN, PRO, MET, THR, ASN, GLN, ASN, SER, THR, PHE, VAL, ALA, PRO, ASP, THR |

**References**

[1] R.M. Ramos, I.S. Moreira, Computational Alanine Scanning Mutagenesis— An Improved Methodological Approach for Protein–DNA Complexes, J. Chem. Theory Comput. 9 (2013) 4243–4256.

[2] R. Kumari, R. Kumar, O.S.D.D. Consortium, A. Lynn, g_mmpbsa— A GROMACS tool for high-throughput MM-PBSA calculations, J. Chem. Inf. Model. 54 (2014) 1951–1962.

[3] A. Srivastava, P. Rawat, P. Tandon, R.N. Singh, A computational study on conformational geometries, chemical reactivity and inhibitor property of an alkaloid bicuculline with γ-aminobutyric acid (GABA) by DFT, Comput. Theor. Chem. 993 (2012) 80–89.

[4] H.K. Srivastava, G.N. Sastry, Efficient estimation of MMGBSA-based BEs for DNA and aromatic furan amidino derivatives, J. Biomol. Struct. Dyn. 31 (2013) 522–537.
